# Supplementary material for: Genetic analyses of the electrocardiographic QT interval and its components identify additional loci and pathways
Source: Nat Commun. 2022 Sep 1;13:5144. doi: 10.1038/s41467-022-32821-z (PMC9436946; doi:10.1038/s41467-022-32821-z)
Supplement: Supplementary file 1 — Supplementary Information [file 41467_2022_32821_MOESM1_ESM.pdf]

Supplementary Figures

A. QT

Multi-Ancestry

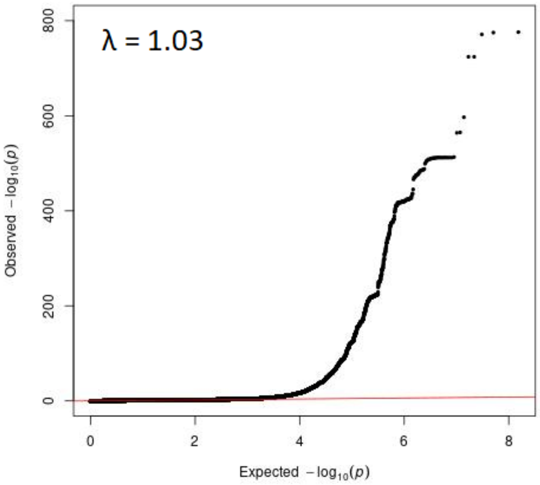

European

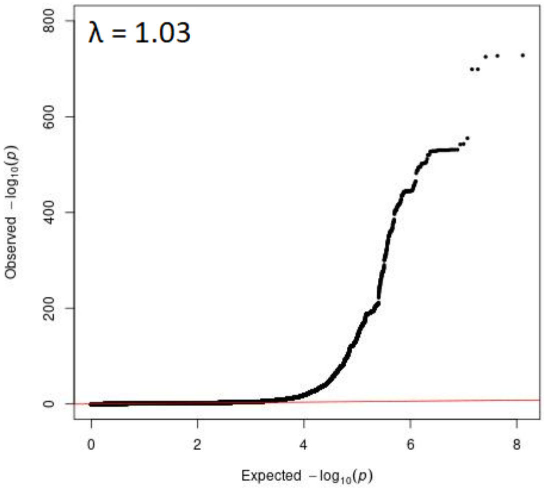

Hispanic

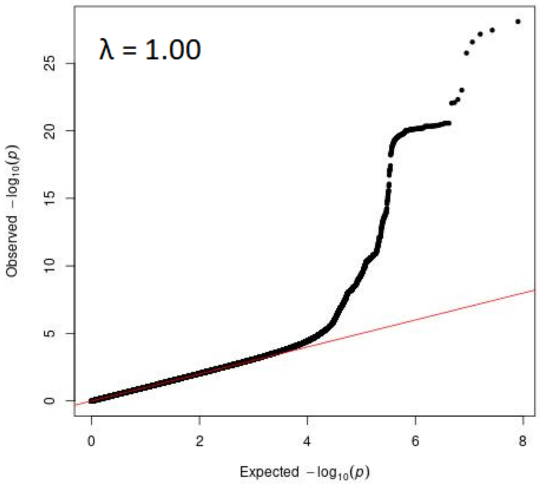

African

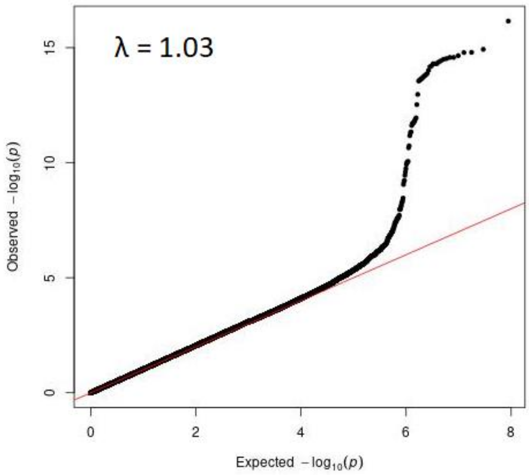

B. JT

Multi-Ancestry

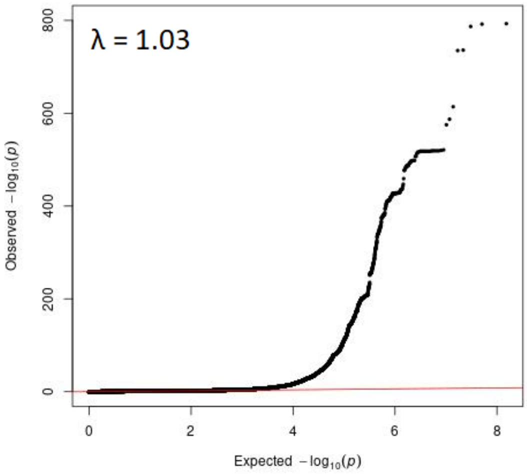

European

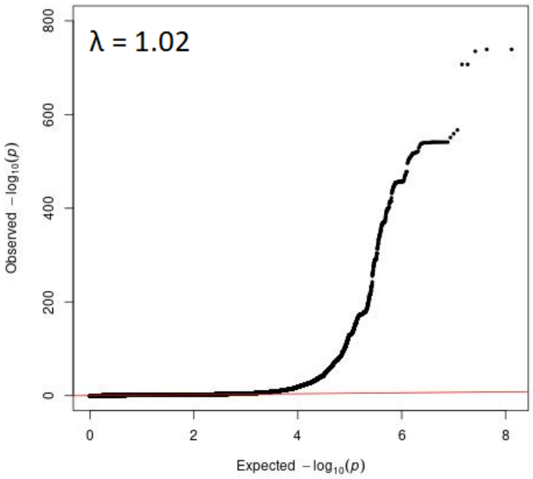

Hispanic

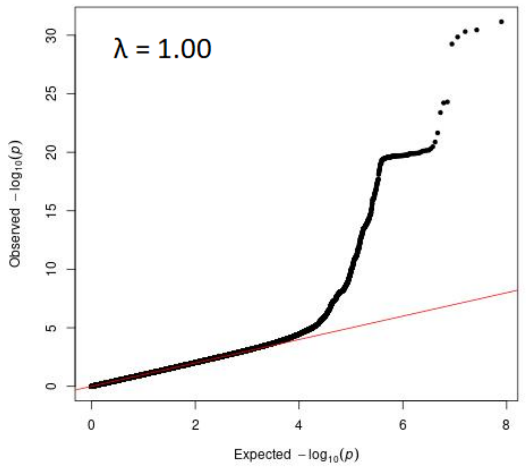

African

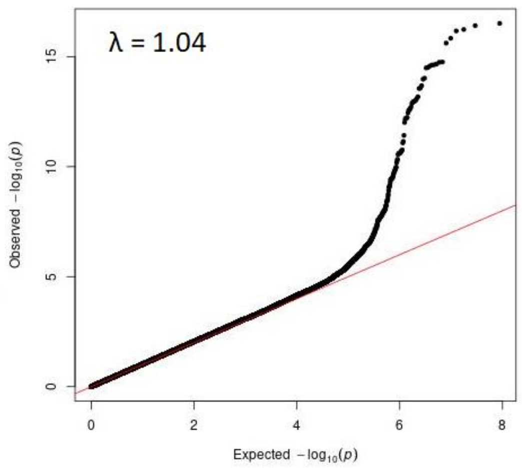

C. QRS

Multi-Ancestry

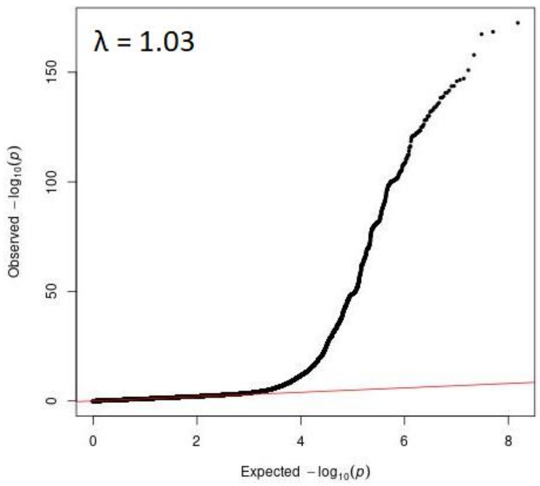

European

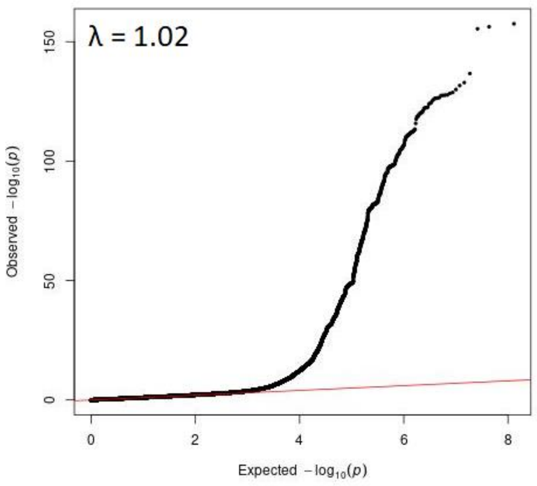

Hispanic

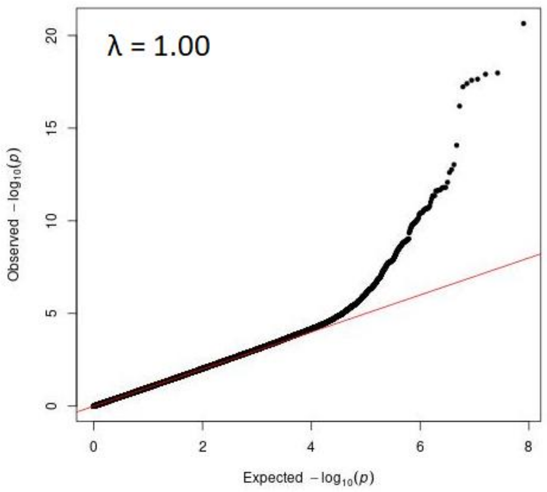

African

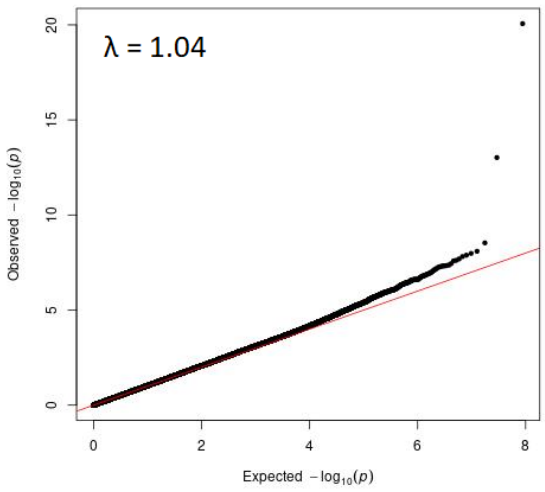

Supplementary Figure 1: QQ plots for QT, JT and QRS meta-analysis
The primary analysis was multi-ancestry. Plots for ancestry-specific meta-analyses (secondary analyses) are also shown. λ = genomic control lambda. *p* = *P*-value. A) QT meta-analysis, B) JT C) QRS.

A. QT Multi-ancestry

FEMALE

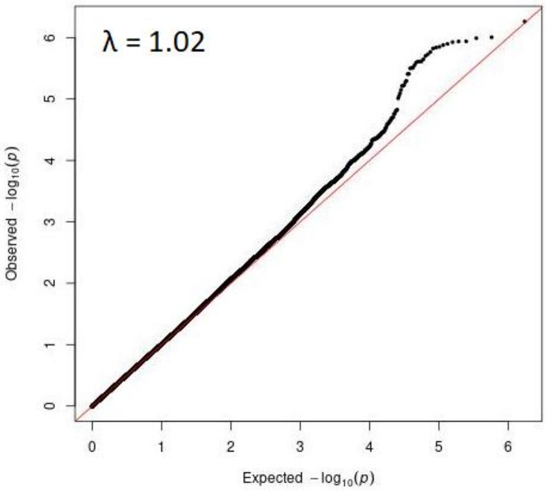

MALE

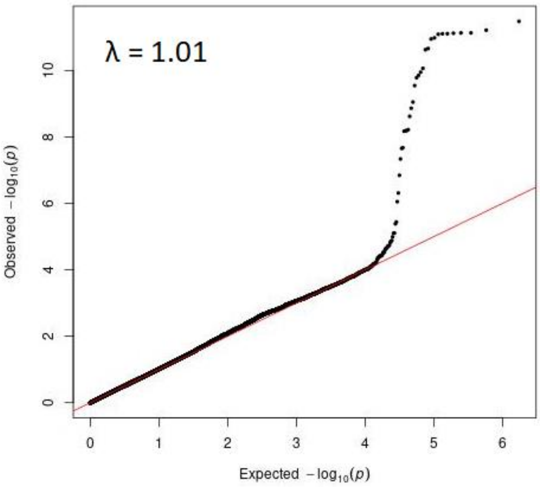

B. **JT Multi-ancestry**

FEMALE

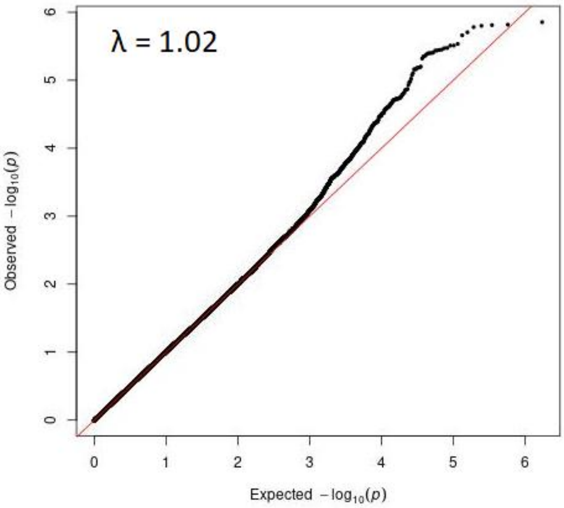

MALE

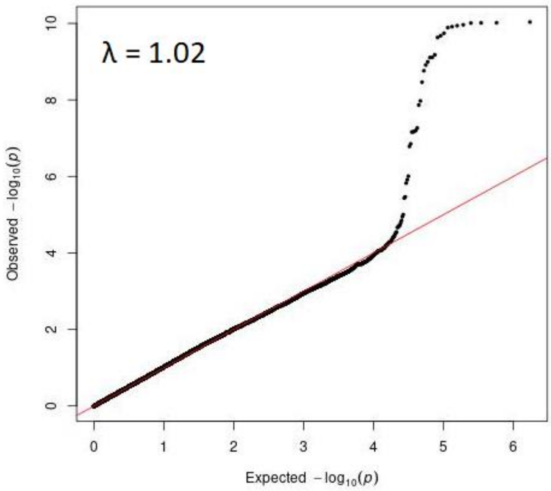

C. QRS Multi-ancestry

FEMALE

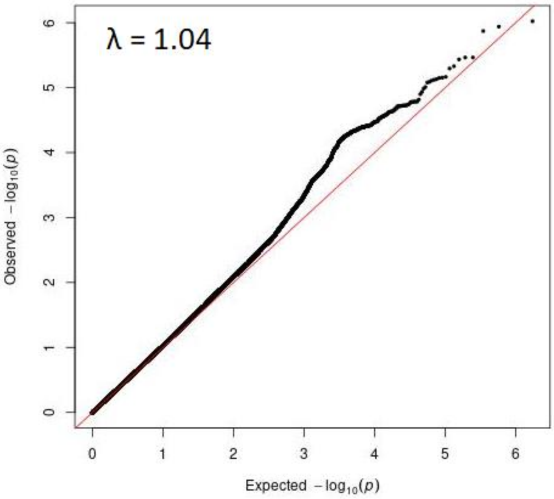

MALE

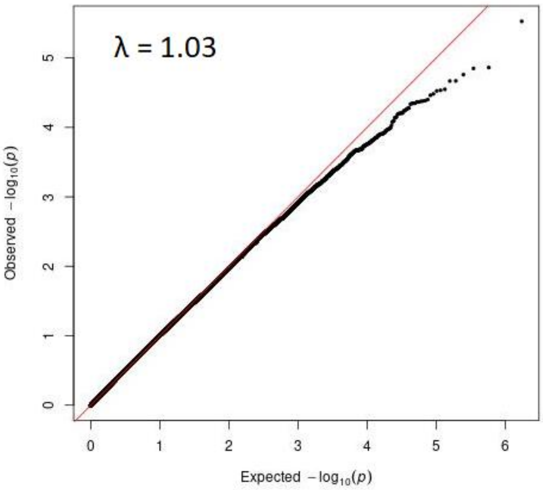

D. QT European

FEMALE | MALE

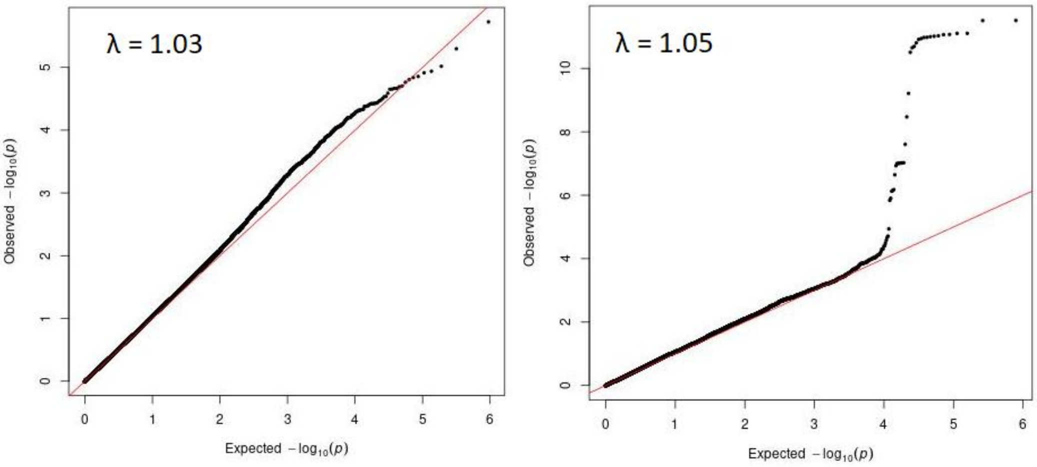

E. JT European

FEMALE | MALE

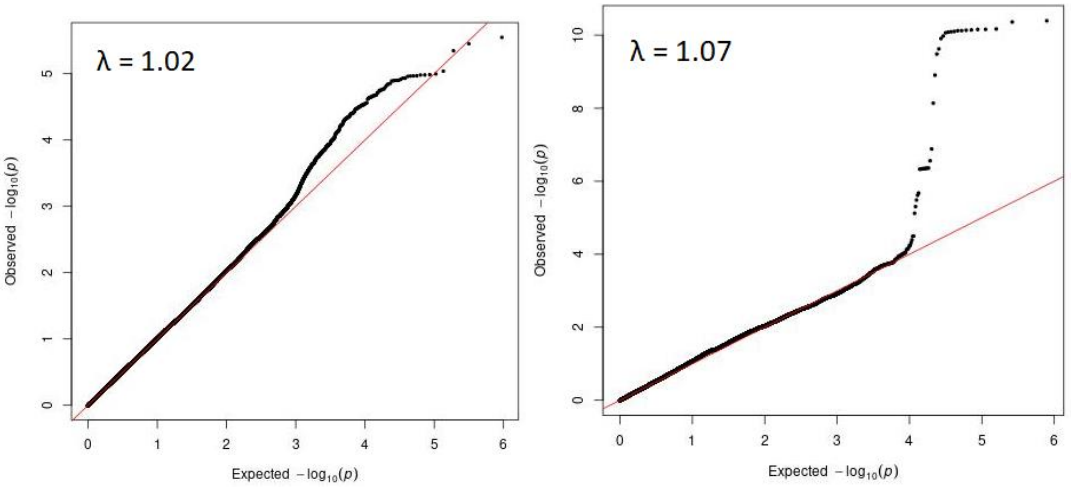

F. QRS European

FEMALE | MALE

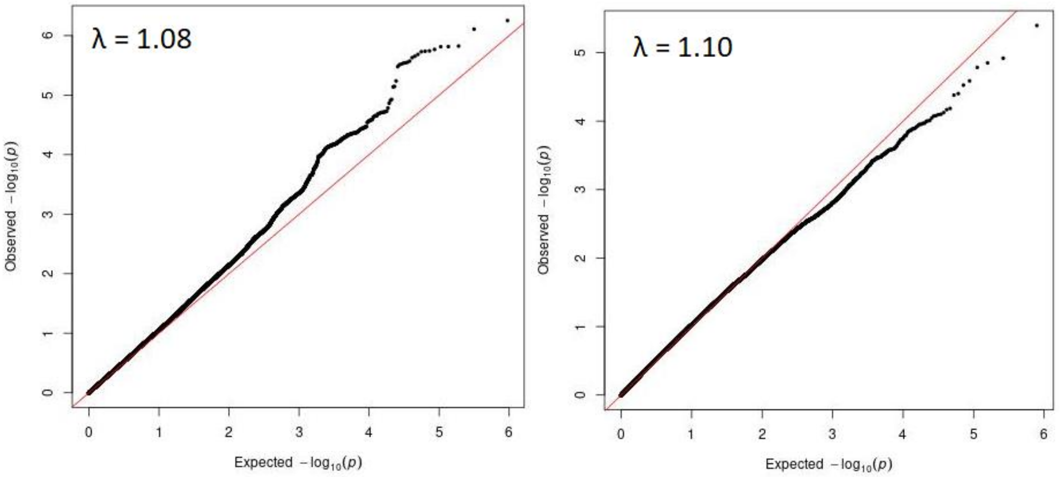

Supplementary Figure 2: QQ plots for multi-ancestry and European X-chromosome analyses

Female and Male stratified results are in left and right panels respectively. λ = genomic control lambda imbedded in each plot (methods). *p* = *P*-value. A) Plots for QT multi-ancestry, B) JT multi-ancestry, c) QRS multi-ancestry D) QT European, E) JT European, F) QRS European meta-analyses.

A. Manhattan plot for QT

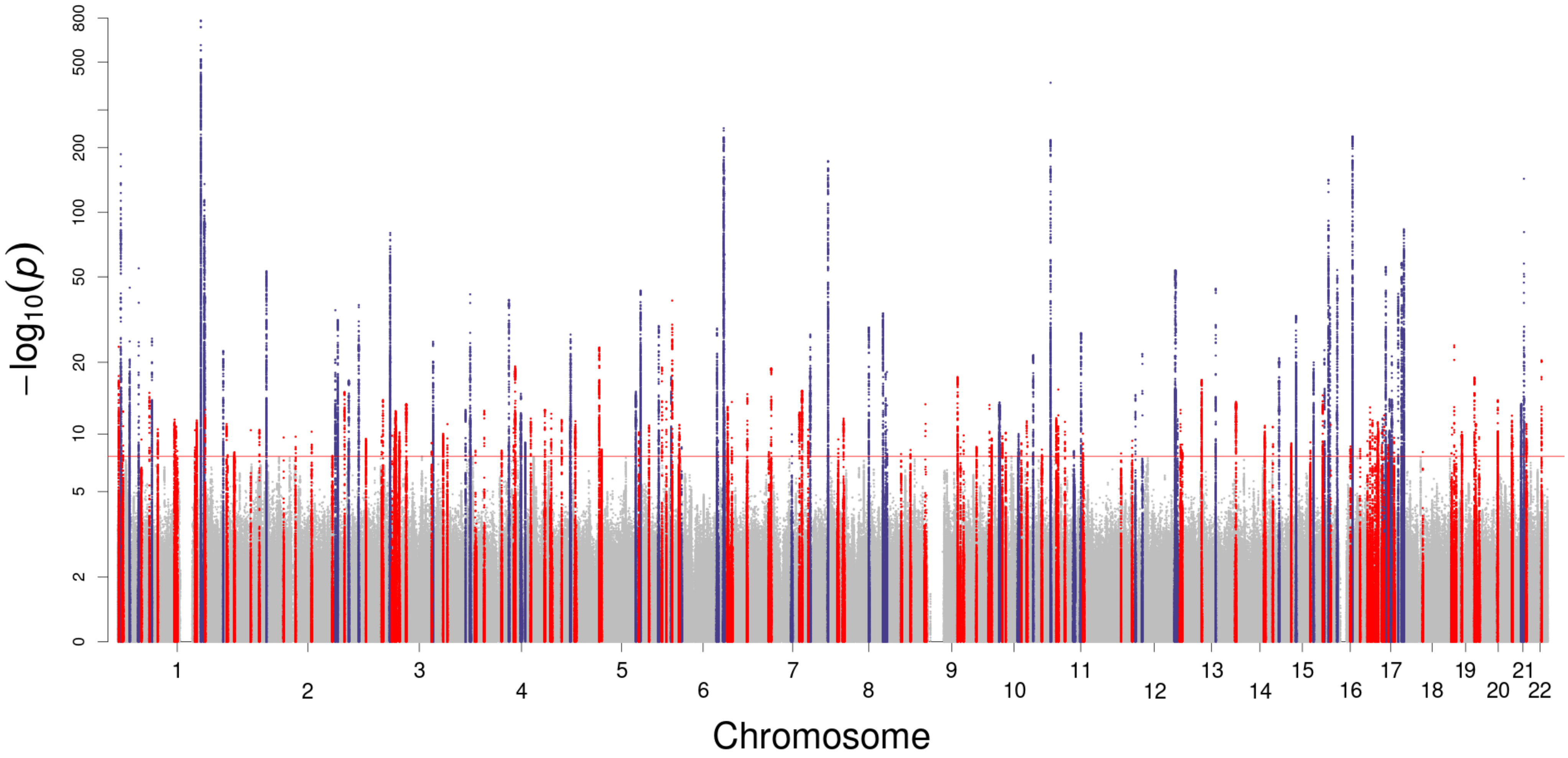

B. Manhattan plot for JT

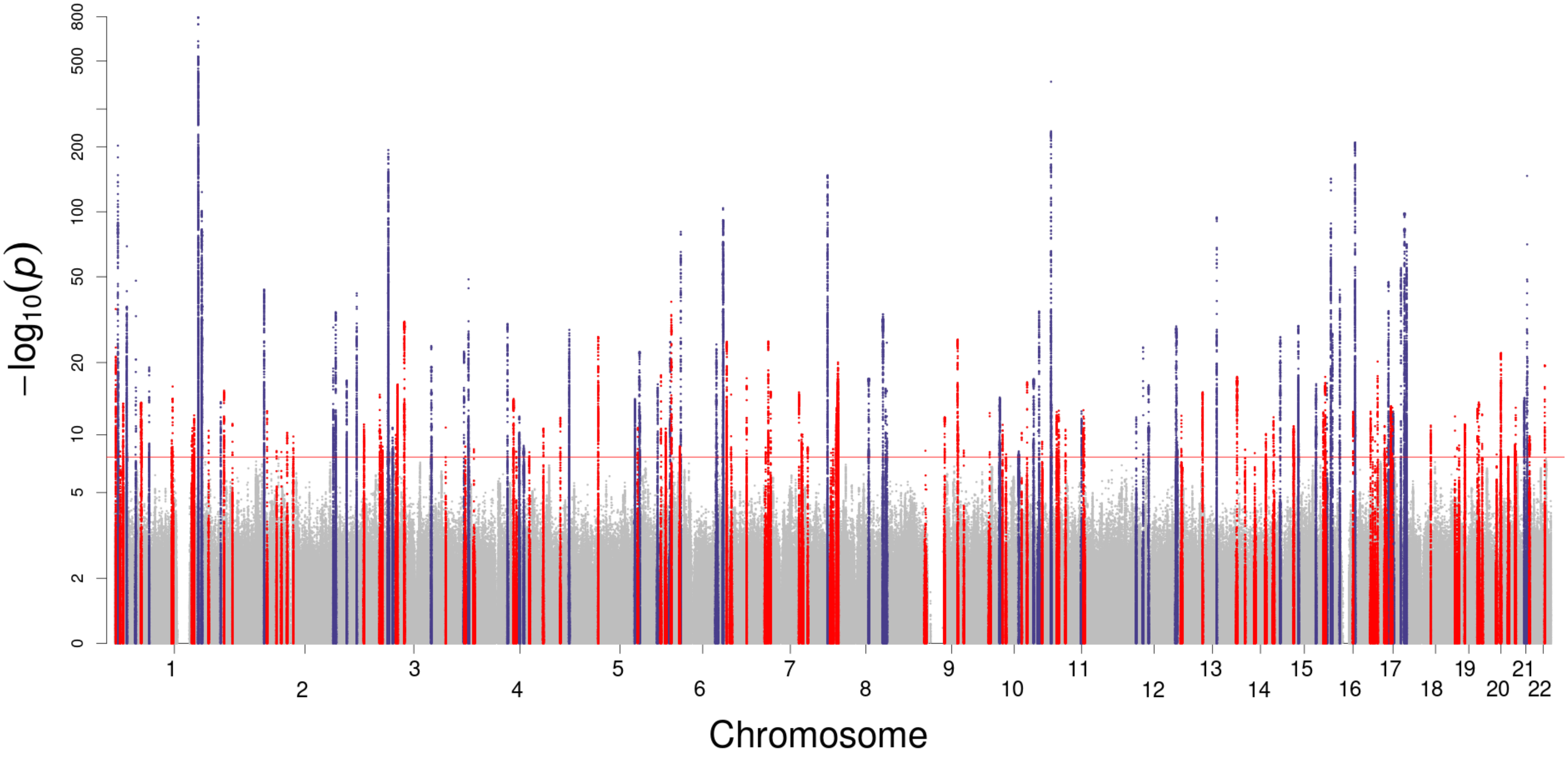

C. Manhattan plot for QRS

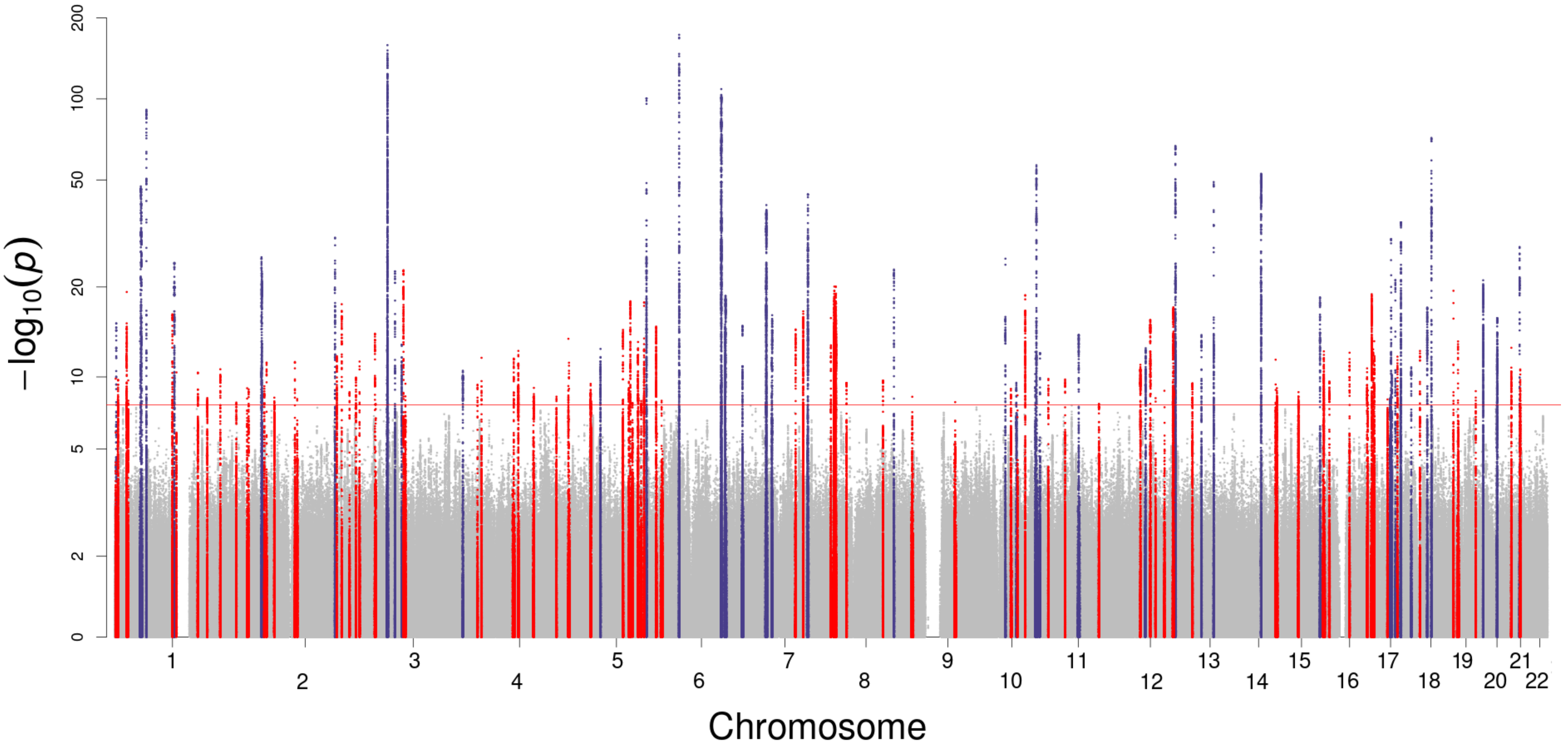

Supplementary Figure 3: Manhattan plots for QT, JT and QRS multi-ancestry meta-analyses

P-values from genome-wide association study meta-analyses. Study level linear regression summary statistics (two-sided) were meta-analysed using METAL, where effect size estimates were weighted using the inverse of the corresponding standard errors. *P* values are plotted on the $-\log_{10}$ scale (Y axis). A Bonferroni-corrected threshold for multiple testing ($P<5 \times 10^{-8}$) was used to declare genome-wide significance. Variants within the boundaries of previously unreported (N=114 for QT, N = 96 (JT), 77 (QRS)) and previously reported loci (N = 62 for QT, N = 59 (JT), 44 (QRS)) are plotted in red and dark blue colors respectively. p = *P*-value.

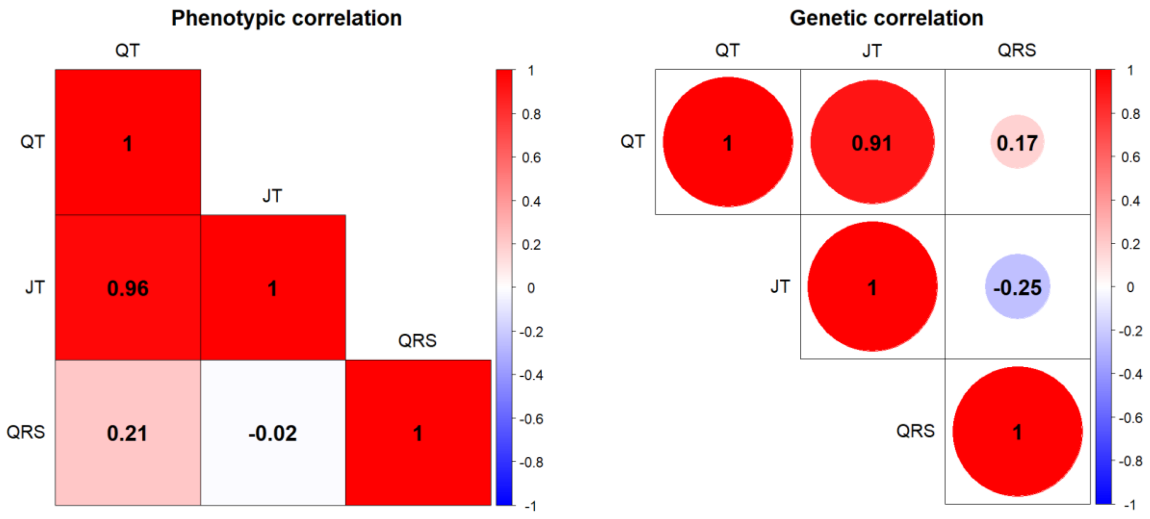

Supplementary Figure 4: Phenotypic and genetic correlations for QT, JT and QRS
Left: Phenotypic correlations (Spearman's rank correlation coefficients [r_s]) were calculated in ~51K UK Biobank individuals of European ancestry. Right: Genetic correlations (r_g) calculated using European ancestry meta-analysis summary statistics with LDSC regression. Positive and negative correlation coefficients are indicated by shading on a scale from bright red (1) to bright blue (-1).

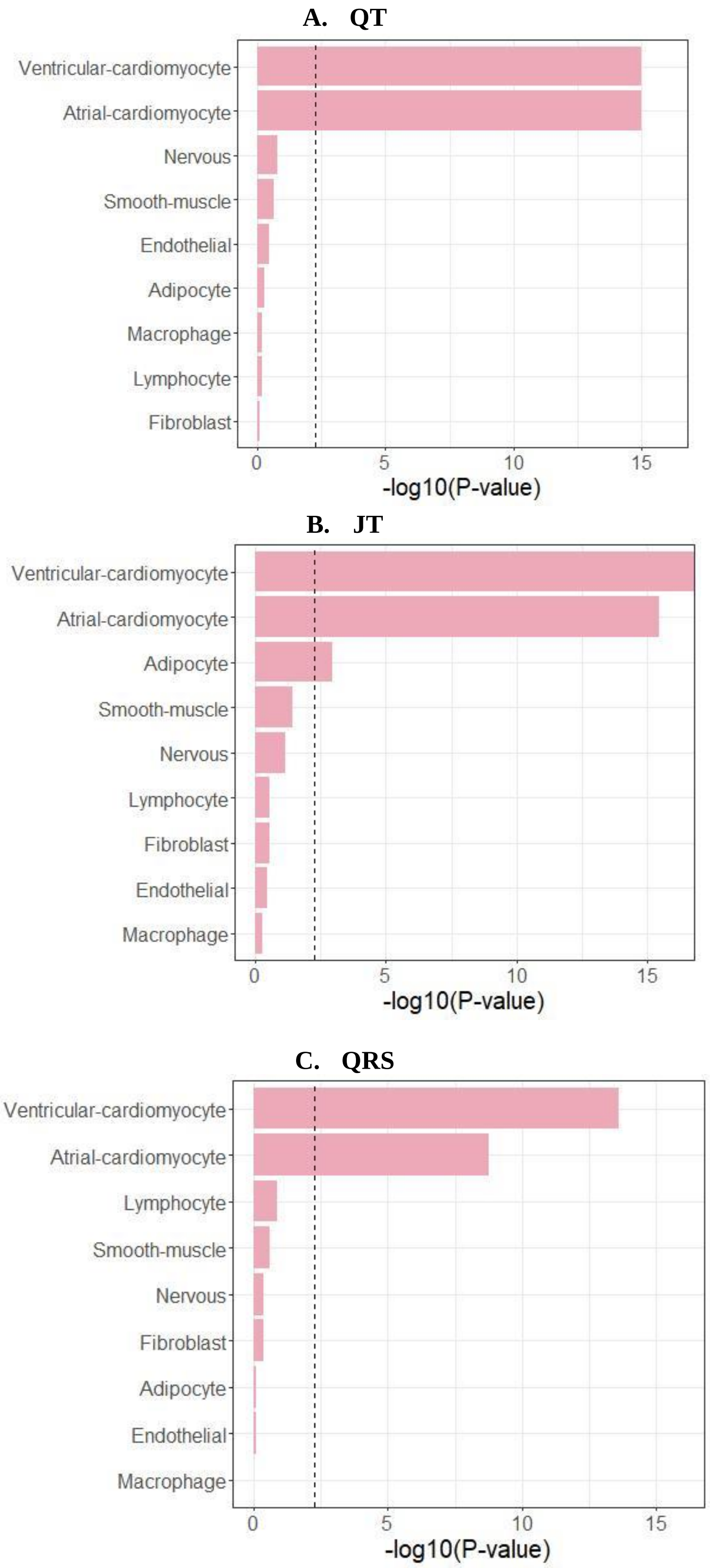

Supplementary Figure 5: Cardiac cell-type overlap with snATAC-seq peaks for QT, JT and QRS
Results for singe nuclear ATAC seq (snATAC-seq) analyses for QT, JT and QRS multi-ancestry meta-analyses. *P*-values were calculated under the null hypothesis (one-sided) that all peak ranks are random (see methods for further information). To correct for multiple testing, a Bonferroni-corrected threshold (P<0.05/number of cell types [9]) to declare significance (vertical dashed line). X-axis: log base 10 *P*-value for cell-type enrichment. Y-axis: Cell-type.

A. QT

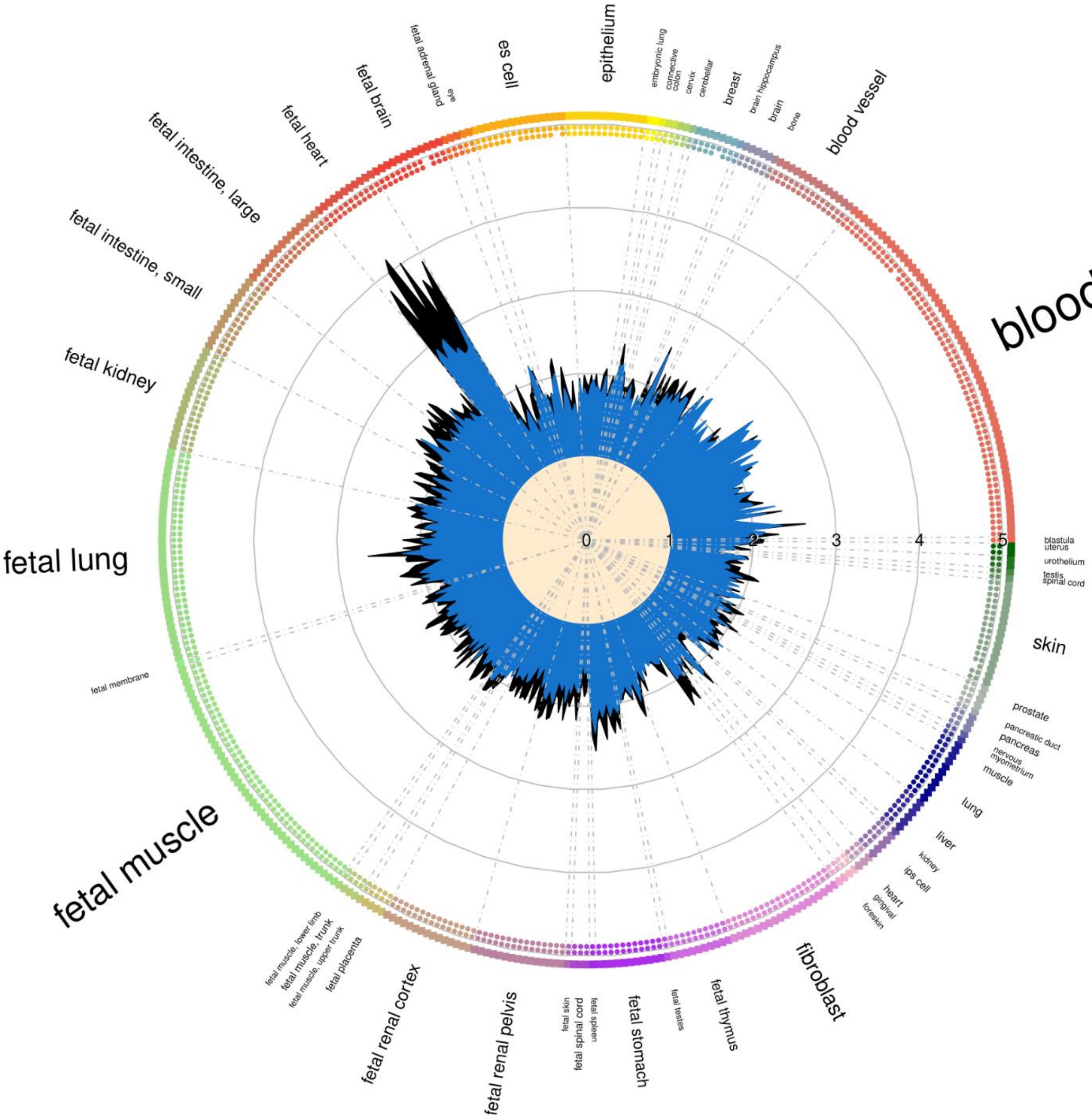
blood
blood vessel
bone
brain
brain hippocampus
breast
cerebellar
cervix
colon
connective
embryonic lung
epithelium
es cell
eye
fetal adrenal gland
fetal brain
fetal heart
fetal intestine, large
fetal intestine, small
fetal kidney
fetal lung
fetal membrane
fetal muscle
fetal muscle, lower limb
fetal muscle, trunk
fetal muscle, upper trunk
fetal placenta
fetal renal cortex
fetal renal pelvis
fetal skin
fetal spinal cord
fetal spleen
fetal stomach
fetal testes
fetal thymus
fibroblast
foreskin
gingival
heart
ips cell
kidney
liver
lung
muscle
myometrium
nervous
pancreas
pancreatic duct
prostate
skin
spinal cord
testis
urothelium
uterus
blastula
0
1
2
3
4
5

B. JT

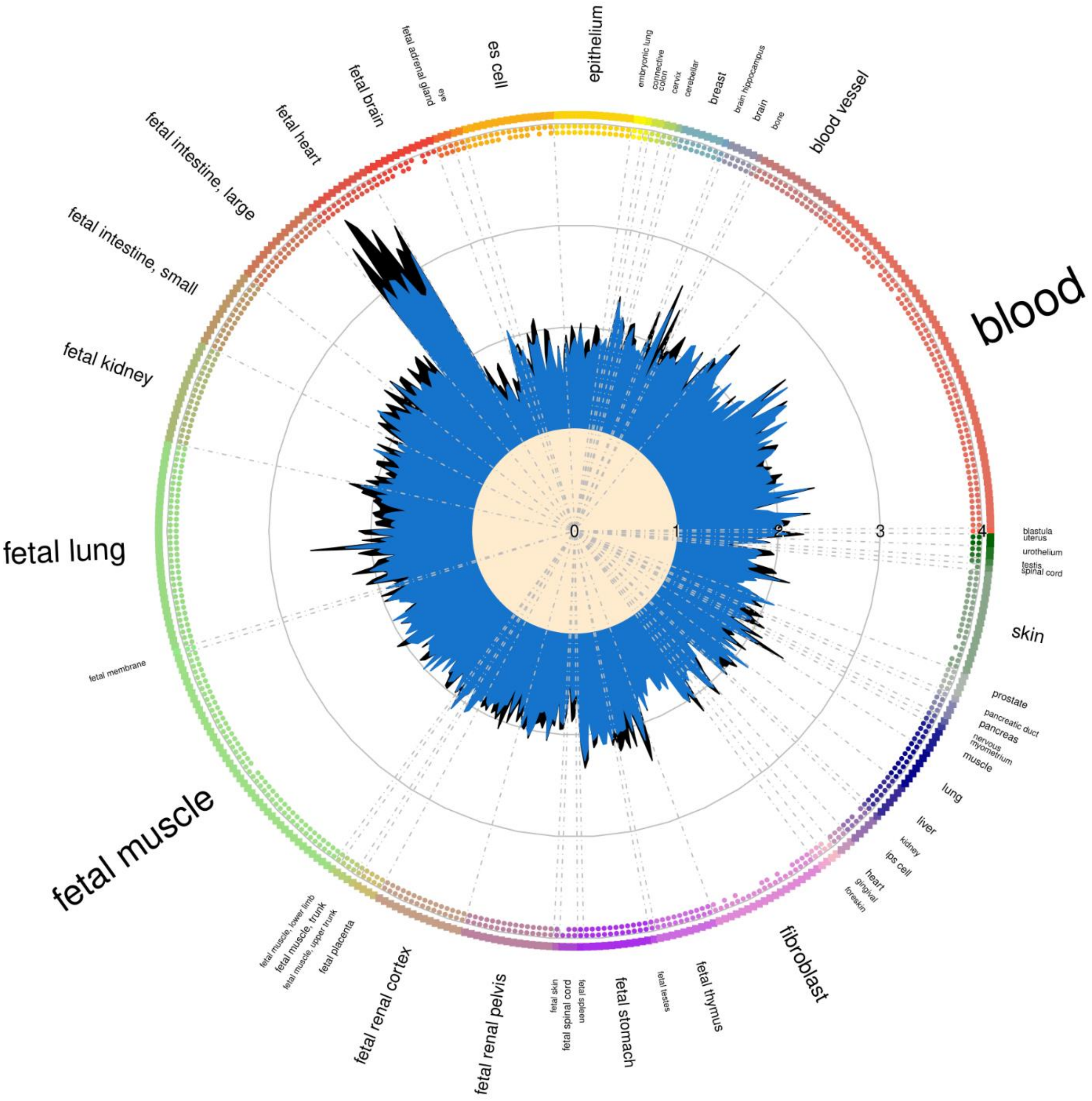

blood
blood vessel
bone
brain
brain hippocampus
breast
cerebellar
cervix
colon
connective
embryonic lung
epithelium
es cell
eye
fetal adrenal gland
fetal brain
fetal heart
fetal intestine, large
fetal intestine, small
fetal kidney
fetal lung
fetal membrane
fetal muscle
fetal muscle, lower limb
fetal muscle, trunk
fetal muscle, upper trunk
fetal placenta
fetal renal cortex
fetal renal pelvis
fetal skin
fetal spinal cord
fetal spleen
fetal stomach
fetal testes
fetal thymus
fibroblast
foreskin
gingival
heart
ips cell
kidney
liver
lung
muscle
myometrium
nervous
pancreas
pancreatic duct
prostate
skin
testis
spinal cord
urothelium
blastula
uterus
0
1
2
3
4

C. QRS

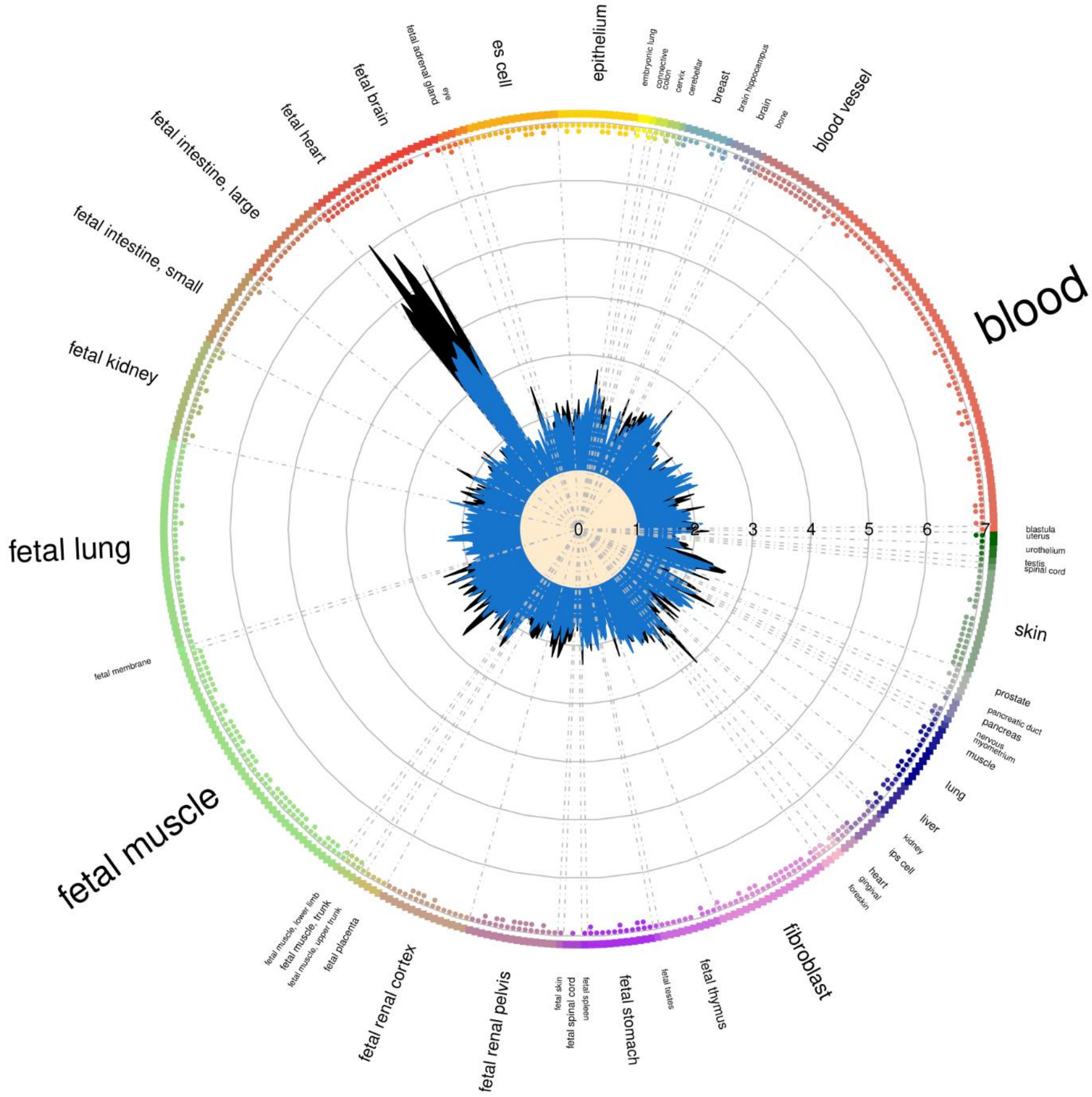

Supplementary Figure 6: Enrichment of QT, JT and QRS variants in DNaseI Hypersensitivity sites
DNaseI hypersensitivity sites were derived from the Encyclopaedia of DNA elements (ENCODE) and Roadmap Epigenomics projects using GARFIELD (Methods). European ancestry meta-analysis results were used. Tissue font size is proportional to the number of cell types for that tissue. Radial plot shows the odds ratio (OR) for enrichment of ECG variants at DNaseI hypersensitivity sites in each cell type. Small dots on the inner side of the outer circle indicate if the observed enrichment was significant at multiple testing GWAS *P*-value thresholds (<1x10^{-5} and < 5x10^{-8}) in the direction outside to inside.

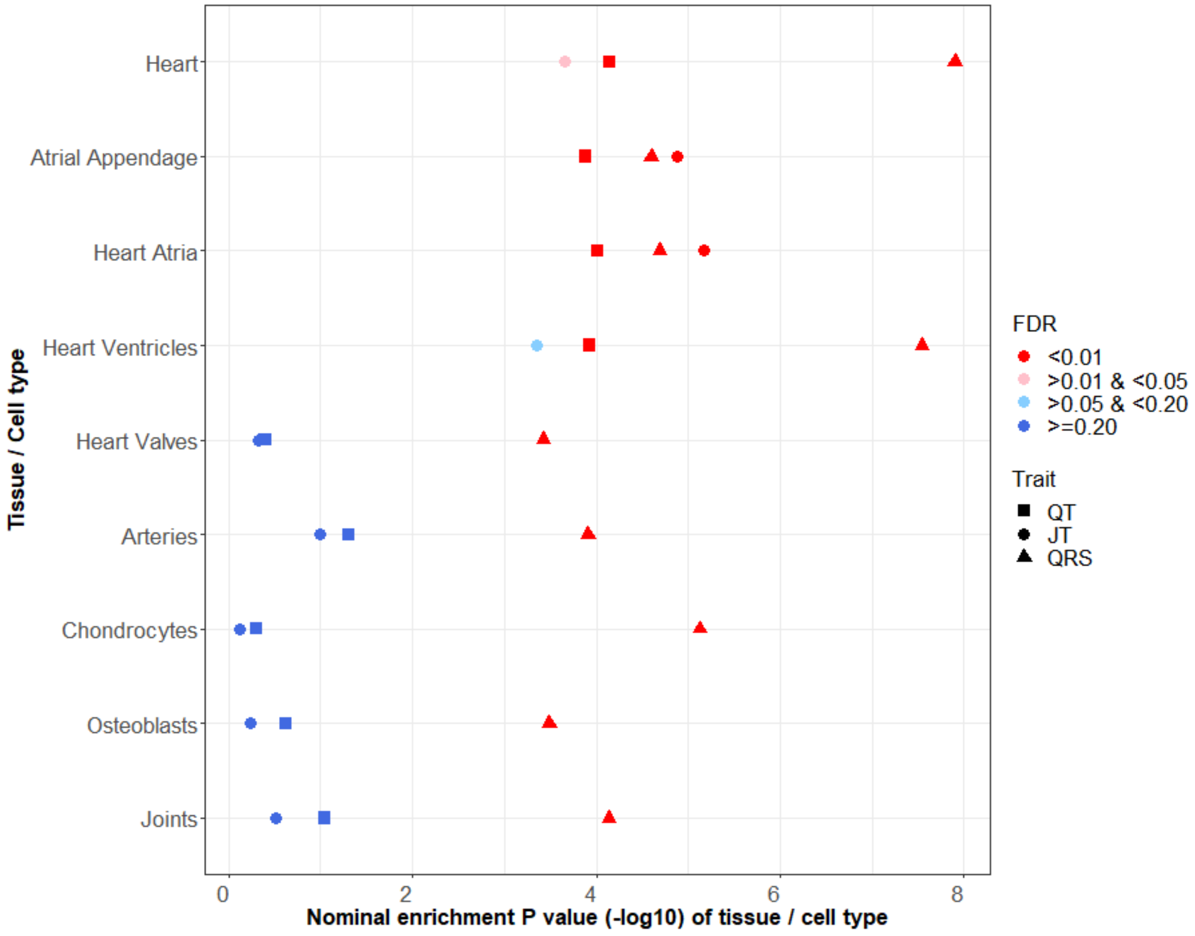

Supplementary Figure 7: DEPICT tissue/cell type enrichment analysis
P-values for enrichment in each tissue / cell-type as output by DEPCIT, which compares z-scores derived from Welch's t-test against the null hypothesis (see methods for further information). Results are color coded according to the false discovery rate (FDR) value (see legend). An FDR <0.01 was used to declare statistically significant findings. X axis – Nominal *P*-value for enrichment, Y axis – Tissue/cell types. Detailed results are reported in Supplementary Data 15.

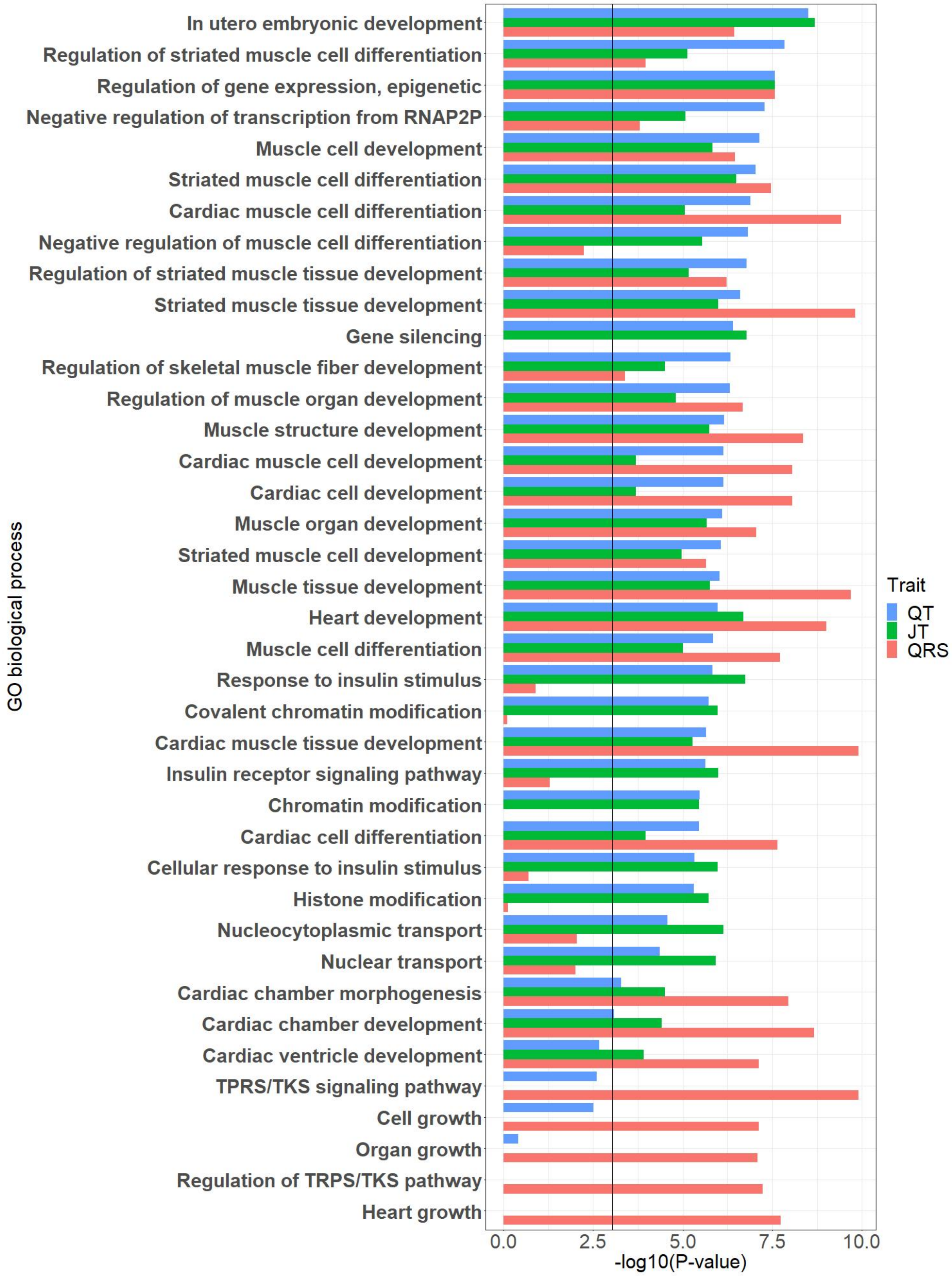

Supplementary Figure 8: Top enriched Gene-Ontology biological processes

Top 20 GO terms (biological processes only) with corresponding -log10 *P*-values for enrichment (X-axis), for QT (shaded blue), JT (green) and QRS (red). *P*-values are computed by DEPCIT by comparing z-scores derived from Welch's t-test against the null hypothesis (see methods for further information). The vertical line indicates the P-value threshold corresponding to a false discovery rate <0.01, which as used to declare statistically significant findings. Full results can be found in Supplementary Data 16. TRPS/TKS: transmembrane receptor protein serine/threonine kinase, RNAP2P: RNA polymerase II promoter.

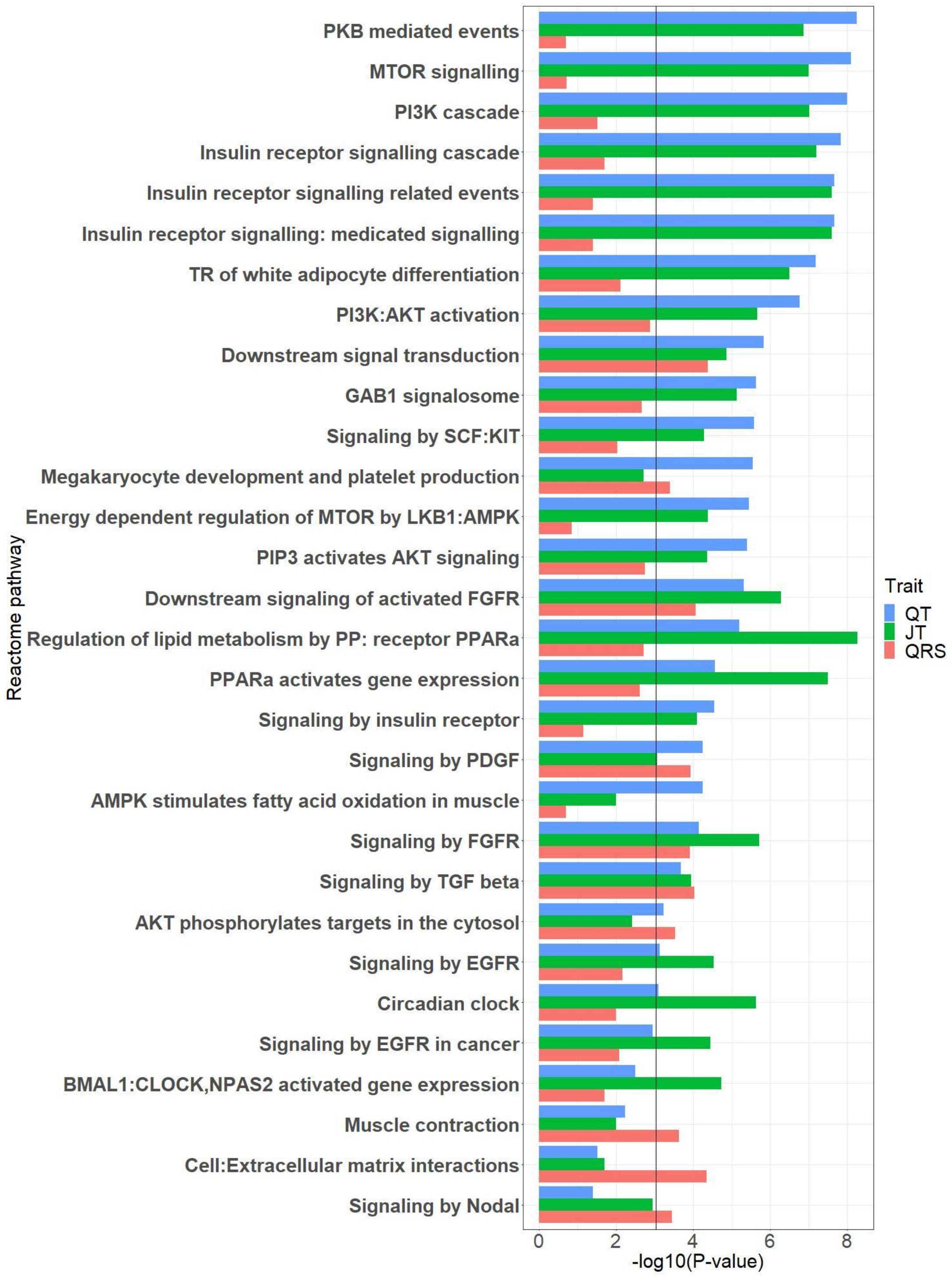

Supplementary Figure 9: Top enriched Reactome pathways

Top 20 Reactome pathways for QT and JT, and top 11 for QRS (only 11 had a false discovery rate <0.01), with -log10 *P*-values for enrichment (X-axis), for QT (shaded blue), JT (green) and QRS (red). *P*-values are computed by DEPCIT by comparing z-scores derived from Welch's t-test against the null hypothesis (see methods for further information). The vertical line indicates the P-value threshold corresponding to a false discovery rate <0.01, which as used to declare statistically significant findings. Full results can be found in Supplementary Data 16. AKT: Protein kinase B, EGFR: Epidermal growth factor receptor, AMPK: AMP-activated protein kinase, FGFR: Fibroblast growth factor receptor, GAB1: GRB2-associated-binding protein 1, KIT: receptor Kit, LKB1: liver kinase B1, MTOR: mammalian target of rapamycin, PI3K: Phosphatidlyinositol-3-kinase, PDGF: Platelet-derived growth factor, PKB: Protein kinase B, PP: peroxisome proliferator, PPARa: Peroxisome proliferator-activated receptor alpha, PIP3: Phosphatidylinositol-3,4,5-triphosphate, SCF: Stem cell factor, TGF: Transforming growth factor, TR: Transcriptional regulation.

Supplementary Notes

Supplementary Note 1 – Study information for genome-wide association study

ARIC - Atherosclerosis Risk in Communities

The Atherosclerosis Risk in Communities (ARIC) Study is a prospective community-based study of cardiovascular disease and its risk factors. At baseline (1987-89), 15,792 men and women age 45-64 were recruited from 4 communities in the US (Washington County, Maryland; Forsyth County, North Carolina; Jackson, Mississippi; Minneapolis suburbs, Minnesota). Participants were mostly white in the Minnesota and Washington County field centers, white and African American in Forsyth County, and exclusively African American in the Jackson field center. Cohort members completed five clinic examinations, conducted approximately three years apart between 1987 and 1998, with a fifth visit conducted from 2011 – 2013. Clinic examinations included assessment of cardiovascular risk factors, self-reported medical family history, employment and educational status, diet, physical activity, comorbidity, clinical and laboratory measurements. The present analyses utilized ECG measurements from the baseline assessment.

Digital 12-lead ECGs were obtained using MAC PC Personal Cardiographs (Marquette Electronics Inc., Milwaukee, WI) and were subsequently submitted to a central reading center at the EPICORE Center (University of Alberta, Edmonton, Alberta, Canada) and thereafter to the Epidemiological Cardiology Research Center (EPICARE), Wake Forest University, Winston-Salem, NC. All ECGs were visually inspected for quality and legibility at their acquisition and by the reading centers.

For assessment of QT interval, participants were asked not to smoke or ingest caffeine for at least 1 hour prior to the ECG being obtained. After resting for 5-10 minutes while the electrodes were being placed, a standard supine 12-lead electrocardiogram and a 2-minute paper recording of a three-lead (leads V1, II, and V5) rhythm strip were made. The QT interval from the digital 12-lead ECG was determined by identifying Q-onset and T-wave offset in all three leads. T-wave offset was defined as the point of maximum change of slope as the T-wave merges with the baseline as implemented in the Novacode ECG measurement and classification program as has been described in detail (https://pubmed.ncbi.nlm.nih.gov/9682893/) and used in prior ARIC studies of the QT interval (https://pubmed.ncbi.nlm.nih.gov/14975464/). U-waves were not detected by the Novacode algorithm. QRS and JT interval were measured automatically from baseline ECGs.

Bambui - Brazilian Bambuí Cohort Study of Ageing

A cohort study designed to identify predictors of adverse health events in the elderly. The study population comprises all residents of Bambuí (Minas Gerais, Brazil), aged 60 or more years (n=1.742) at the baseline in 1997. From these, 92.2% were interviewed and 85.9% underwent clinical examination, consisting of hematological and biochemical tests, serology for Trypanosoma cruzi, anthropometric and blood pressure measures and electrocardiogram. Cohort members undergo annual follow-up visits, which consist of an interview and verification of death certificates. Other procedures were repeated in selected years (2000, 2002 and 2008). From 1997 to 2007, during a mean follow-up of 8.6 years, 641 participants died and 96 (6.0%) were lost to follow-up.

BioMe - The IPM Bio*Me* Biobank

The Mount Sinai BioMe Biobank, founded in September 2007, is an ongoing, broadly consented EHR-linked bio- and data repository that enrolls participants non-selectively from the Mount Sinai Medical Center patient population. The BioMe Biobank draws from a population of over 70,000 inpatient and 800,000 outpatient visits annually from over 30 broadly selected clinical sites of the Mount Sinai Medical Center (MSMC). As of September 2017, BioMe has enrolled more than 42,000 patients that represent a broad racial, ethnic and socioeconomic diversity with a distinct and population-specific disease burden, characteristic of the communities served by Mount Sinai Hospital. BioMe participants are predominantly of African (AA, 24%), Hispanic/Latino (HL, 35%), European (EA, 32%), and other ancestry (OA, 10%). The BioMe Biobank Program operates under a Mount

Sinai Institutional Review Board-approved research protocol. All study participants provided written informed consent.

BRIGHT - British Genetics of Hypertension
Participants of the BRIGHT Study were recruited from the Medical Research Council General Practice Framework and other primary care practices in the UK. Each case had a history of hypertension diagnosed prior to 60 years of age with confirmed blood pressure recordings corresponding to seated levels >150/100mmHg (1 reading) or mean of 3 readings >145/95 mmHg. BRIGHT is focused on recruitment of hypertensive individuals with BMI<30. Sample selection for GWAS was based on DNA availability and quantity.

Broad AF
The Broad AF Study is a collaborative project to investigate the genetic determinants of atrial fibrillation (AF), comprised of 17,517 AF cases and 10,987 referents from 26 studies. Details of study description, genotyping, and imputation were described previously. 2 Briefly, genetic variants were centrally genotyped on the Infinium PsychArray-24 v1.2 Bead Chip, and jointly 8 called and quality controlled at the Broad Institute. After pre-imputation quality control, variants were imputed using the 1000 Genomes reference panel. A total of 3,461 individuals free of AF met the inclusion criteria of the current study and were included in the PR analysis. Individuals from the following participating studies were included: Vanderbilt University Medical Center Biobank (BioVU), Australian Familial AF Study, Danish AF Study, Groningen Genetics of Atrial Fibrillation (GGAF), Genetic Risk Assessment of Defibrillator Events Study (GRADE), Malmö Preventive Project (MPP-AF, and MPP-Echo), and Intermountain INSPIRE Registry. Study details were previously reported.

CAMP-MGH - MGH Cardiology and Metabolic Patient Cohort
The MGH Cardiology and Metabolic Patient Cohort is comprised of 3850 subjects recruited from the ambulatory MGH Cardiology Practice between 2009 and 2012.

CHRIS - The Cooperative Health Research in South Tyrol study
The Cooperative Health Research In South Tyrol (CHRIS) study is a population-based study with a longitudinal lookout to investigate the genetic and molecular basis of age-related common chronic conditions and their interaction with life style and environment in the general population. The study was approved by the Ethics Committee of the Autonomous Province of Bolzano.

CHS - Cardiovascular Health Study
The Cardiovascular Health Study (CHS) is a population-based cohort study of risk factors for coronary heart disease and stroke in adults ≥65 years conducted across four field centers . The original predominantly European ancestry cohort of 5,201 persons was recruited in 1989-1990 from random samples of the Medicare eligibility lists; subsequently, an additional predominantly African-American cohort of 687 persons was enrolled in 1992-1993 for a total sample of 5,888.

Blood samples were drawn from all participants at their baseline examination and DNA was subsequently extracted from available samples. Genotyping was performed at the General Clinical Research Center's Phenotyping/Genotyping Laboratory at Cedars-Sinai among CHS participants who consented to genetic testing and had DNA available using the Illumina 370CNV BeadChip system (for European ancestry participants, in 2007) or the Illumina HumanOmni1-Quad_v1 BeadChip system (for African-American participants, in 2010).

CHS was approved by institutional review committees at each field center and individuals in the present analysis had available DNA and gave informed consent including consent to use of genetic information for the study of cardiovascular disease.

ERF - Erasmus Rucphen Family Study
The Erasmus Rucphen Family study is comprised of a family-based cohort embedded in the Genetic Research in Isolated Populations (GRIP) program in the southwest of the Netherlands. The aim of this

program is to identify genetic risk factors for the development of complex disorders. In ERF, twenty-two families that had a large number of children baptized in the community church between 1850 and 1900 were identified with the help of detailed genealogical records. All living descendants of these couples, and their spouses, were invited to take part in the study. Comprehensive interviews, questionnaires, and examinations were completed at a research center in the area; approximately 3,200 individuals participated. Examinations included 12 lead ECG measurements. Electrocardiograms were recorded on ACTA electrocardiographs (ESAOTE, Florence, Italy) and digital measurements of the QRS and QT intervals were made using the Modular ECG Analysis System (MEANS). Data collection started in June 2002 and was completed in February 2005. In the current analyses, 2442 participants for whom complete phenotypic, genotypic and genealogical information was available were studied.

FINCAVAS - Finnish Cardiovascular Study
The purpose of the Finnish Cardiovascular Study (FINCAVAS) is to construct a risk profile - using genetic, hemodynamic and electrocardiographic (ECG) markers - of individuals at high risk of cardiovascular diseases, events and deaths. All patients scheduled for an exercise stress test at Tampere University Hospital and willing to participate have been recruited between October 2001 and December 2007. The final number of participants is 4,567. In addition to repeated measurement of heart rate and blood pressure, digital high-resolution ECG at 500 Hz was recorded continuously during the entire exercise test, including the resting and recovery phases. About 20% of the patients are were examined with coronary angiography. Genetic variations known or suspected to alter cardiovascular function or pathophysiology are were analyzed to elucidate the effects and interactions of these candidate genes, exercise and commonly used cardiovascular medications.

GAPP - Genetic and phenotypic determinants of blood pressure and other cardiovascular risk factors
GAPP is a population-based prospective cohort study involving a representative sample of healthy adults aged 25-41 years residing in the Principality of Liechtenstein. Exclusion criteria were the presence of cardiovascular disease, diabetes, obstructive sleep apnea and a body mass index >35kg/m2. A standardized 12-lead ECG was obtained in all participants.

GESUS - The Danish General Suburban Population Study
GESUS is a population-based prospective cohort study from Naestved Municipality (70km south of Copenhagen), Denmark. The study enrolled 21,205 adult participants between 2010-2013. Age was 20 years or above (20-100 years). All participants answered a questionnaire and had a physical examination (including EKG, laboratory tests, anthropometrics, biological samples for biobank etc.) performed at the Department of Clinical Biochemistry, Naestved Hospital, Denmark. A standard 12-lead paper ECG was obtained in all participants, and a corresponding electronic ECG was obtained in 8939 participants. ECG information was obtained from the MUSE Cardiology Information System (GE Healthcare, Wauwatosa, Wisconsin, USA) and analyzed by Marquette 12SL algorithm version 21. The ECGs were recorded with a sample rate of 500 Hz and a resolution of 4.88 μV per least significant bit. At the time of this study, 3004 participants were genotyped and included.

GS20 - Generation Scotland: Scottish Family Health Study
The GS:SFHS study recruited 23,960 participants aged 18–100 years between 2006–11; full details are reported elsewhere [Smith et al, IJE 2012]. Participants came from across Scotland, with some family members from further afield. The sample was 59% female, with a wide range of ages and socio-demographic characteristics. Most (87%) participants were born in Scotland and 96% in the UK or Ireland.

HCHS/SOL - Hispanic Community Health Survey/Study of Latinos
The Hispanic Community Health Study/ Study of Latinos (HCHS/SOL) The HCHS/SOL is a multicenter, community-based cohort study of U.S. Hispanics/Latinos. Goals of the study are to examine the prevalence of and risk factors for several disorders including heart, lung, blood, and kidney phenotypes. HCHS/SOL investigators sampled 16,415 males and females aged 18-74 years at

baseline from four study communities: The Bronx, NY, Chicago, IL, Miami, FL, and San Diego, CA. HCHS/SOL recruitment centers were selected so that the study would include at least 2,000 participants in each of the following designations: Mexican, Puerto Rican, Dominican, Cuban, and Central and South American.

Health ABC - Health, Aging, and Body Composition Study

The Health Aging and Body Composition (Health ABC) Study is a NIA-sponsored cohort study of the factors that contribute to incident disability and the decline in function of healthier older persons, with a particular emphasis on changes in body composition in old age. Between 4/15/97 and 6/5/98 the Health ABC study has recruited 3,075 70-79 year old community-dwelling adults (41% African-American), who were initially free of mobility and activities of daily living disability. The key components of Health ABC include a baseline exam, annual follow-up clinical exams, and phone contacts every 6 months to identify major health events and document functional status between clinic visits. Provision has been made for banking of blood specimens and extracted DNA (HealthABC repository).

INGI-CAR - INGI-CARLANTINO

INGI-CAR consisted of about 1000 subjects who were drawn from Carlantino, an isolated village of southern Italy. Ethics approval was obtained from the Ethics Committee of the IRCCS Burlo Garofolo in Trieste. Written informed consent was obtained from every participant of the study. The study population had undergone clinical and instrumental evaluations between 1998 and 2005. For all subjects, anthropometrics variables (such as height, weight, etc.) were taken and a structured questionnaire about lifestyle and medical history was filled out. In addition, blood pressure, body-mass index, biochemical analyses, ECG and cardiovascular evaluation were collected.

INGI-FVG - INGI-Friuli Venezia Giulia

The INGI-FVG cohort consisted of about 1700 subjects drawn from the project "Genetic Park of Friuli Venezia Giulia". This study examined 6 isolated villages in the North-East of Italy between 2008 and 2010. Ethics approval was obtained from the Ethics Committee of the IRCCS Burlo Garofolo in Trieste. Written informed consent was obtained from every participant of the study. The study population had undergone clinical and instrumental evaluations. For all subjects, anthropometrics variables (such as height, weight, etc) were taken and a structured questionnaire about lifestyle and medical history was filled out. In addition, blood pressure, body-mass index, biochemical analyses, ECG and cardiovascular evaluation were collected.

INTER99 - Inter99

The Inter99 study carried out in 1999-2001 included invitation of12934 persons aged 30-60 years drawn from an age- and sex-stratified random sample of the population (16). The baseline participation rate was 52.5%, and the study included 6784 persons. The Inter99 study was a population-based randomized controlled trial (CT00289237, ClinicalTrials.gov) and investigated the effects of lifestyle intervention on CVD. Here 5827 participants with information on lipids and exome chip were analyzed. ECG information was obtained from the MUSE Cardiology Information System (GE Healthcare, Wauwatosa, Wisconsin) analyzed by Marquette 12SL algorithm version 21.

JHS - Jackson Heart Study

The JHS is a single-site cohort study of 5,306 extensively phenotyped African American women and men. Three clinical examinations have been completed, including the baseline examination, Examination 1 (2000–2004), Examination 2 (2005–2008), and Examination 3 (2009–2013), allowing comprehensive assessment of cardiovascular health and disease of the cohort at approximately four-year intervals. Ongoing monitoring of hospitalizations for cardiovascular events (coronary heart disease, heart failure and stroke) and deaths among cohort participants are accomplished by annual telephone follow-up interviews, surveillance of hospital discharge records (since 2000 for coronary heart disease and stroke, and since 2005 for heart failure), and vital records.

KORA F3/S4 - Kooperative Gesundheitsforschung in der Region Augsburg F3/S4

The KORA study is a series of independent population-based epidemiological surveys of participants living in the city of Augsburg, Southern Germany, or the two adjacent counties. All survey participants are residents of German nationality identified through the registration office and aged between 25 and 74 years at recruitment. KORA F3: The baseline survey KORA S3 was conducted in the years 1994/95. 3,006 participants from KORA S3 were reexamined in a 10-year follow-up (KORA F3) in the years 2004/05. KORA S4: All survey participants are residents of German nationality identified through the registration office and aged between 25 and 74 years at recruitment. The baseline survey KORA S4 was conducted in the years 1999-2001.

LIFELINES - LifeLines, a three-generation cohort study and biobank
The LifeLines Cohort Study, and generation and management of GWAS genotype data for the LifeLines Cohort Study is supported by the Netherlands Organization of Scientific Research NWO (grant 175.010.2007.006), the Economic Structure Enhancing Fund (FES) of the Dutch government, the Ministry of Economic Affairs, the Ministry of Education, Culture and Science, the Ministry for Health, Welfare and Sports, the Northern Netherlands Collaboration of Provinces (SNN), the Province of Groningen, University Medical Center Groningen, the University of Groningen, Dutch Kidney Foundation and Dutch Diabetes Research Foundation.

MESA - Multi-Ethnic Study of Atherosclerosis
The Multi-Ethnic Study of Atherosclerosis (MESA) is a study of the characteristics of subclinical cardiovascular disease (disease detected non-invasively before it has produced clinical signs and symptoms) and the risk factors that predict progression to clinically overt cardiovascular disease or progression of the subclinical disease. The cohort is a diverse, population-based sample of 6,814 asymptomatic men and women aged 45-84. Approximately 38 percent of the recruited participants are white, 28 percent African-American, 22 percent Hispanic, and 12 percent Asian (predominantly of Chinese descent). Participants were recruited during 2000-2002 from 6 field centers across the U.S. (at Wake Forest University; Columbia University; Johns Hopkins University; the University of Minnesota; Northwestern University; and the University of California – Los Angeles). All underwent anthropomorphic measurement and extensive evaluation by questionnaires at baseline, followed by 5 subsequent examinations at intervals of approximately 2-4 years. Age and sex were self-reported.

NEO - Netherlands Epidemiology of Obesity
The NEO was designed for extensive phenotyping to investigate pathways that lead to obesity-related diseases. The NEO study is a population-based, prospective cohort study that includes 6,671 individuals aged 45–65 years, with an oversampling of individuals with overweight or obesity. At baseline, information on demography, lifestyle, and medical history have been collected by questionnaires. In addition, samples of 24-h urine, fasting and postprandial blood plasma and serum, and DNA were collected. Genotyping was performed using the Illumina HumanCoreExome chip, which was subsequently imputed to the 1000 genome reference panel. Participants underwent an extensive physical examination, including anthropometry, electrocardiography, spirometry, and measurement of the carotid artery intima-media thickness by ultrasonography. In random subsamples of participants, magnetic resonance imaging of abdominal fat, pulse wave velocity of the aorta, heart, and brain, magnetic resonance spectroscopy of the liver, indirect calorimetry, dual energy X-ray absorptiometry, or accelerometry measurements were performed. The collection of data started in September 2008 and completed at the end of September 2012. Participants are currently being followed for the incidence of obesity-related diseases and mortality.

OOA - Old Order Amish
The Old Order Amish (OOA) subjects included in this study were participants of several studies of cardiovascular health in relatively healthy volunteers from the OOA community of Lancaster County, PA and their family members. The studies were carried out at the University of Maryland as part of the Amish Complex Disease Research Program (ACDRP). The OOA population of Lancaster County, PA immigrated to the Colonies from Western Europe in the early 1700's. All study protocols were approved by the institutional review board at the University of Maryland and participating institutions. Informed consent was obtained from each of the study participants.

ORCADES - Orkney Complex Disease Study
The Orkney Complex Disease Study (ORCADES) is a family-based, cross-sectional study that seeks to identify genetic factors influencing cardiovascular and other disease risk in the isolated archipelago of the Orkney Isles in northern Scotland (McQuillan et al., 2008). Genetic diversity in this population is decreased compared to Mainland Scotland, consistent with the high levels of endogamy historically. 2078 participants aged 16-100 years were recruited between 2005 and 2011, most having three or four grandparents from Orkney, the remainder with two Orcadian grandparents. Fasting blood samples were collected and many health-related phenotypes and environmental exposures were measured in each individual. All participants gave written informed consent and the study was approved by Research Ethics Committees in Orkney and Aberdeen (North of Scotland REC).

PIVUS - Prospective Investigation of the Vasculature of Uppsala Seniors
The Prospective Investigation of Vasculature in Uppsala Seniors study was initiated in 2001 to investigate the predictive power of different measurements of vascular characteristics for future cardiovascular events, and secondary aims included measurements of cardiac and metabolic function, as well as serum biomarkers and levels of environmental pollutants. All individuals aged 70 living in the community of Uppsala in Sweden were deemed eligible for the study. The subjects were selected from the community register and invited in randomized order between April 2001 and June 2004. They received an invitation letter for participation within 2 months of their 70th birthday. Of the 2,025 subjects invited, 1,016 (507 male, 509 female) subjects agreed to participate. The participants were asked to answer a questionnaire about their medical history, smoking habits and regular medication.

PREVEND - Prevention of REnal and Vascular ENd stage Disease
Prevend is an ongoing prospective study investigating the natural course of increased levels of urinary albumin excretion and its relation to renal and cardiovascular disease. Details of the protocol have been described elsewhere.

PROSPER - PROSpective study of pravastatin in the elderly at Risk for vascular disease
All data come from the PROspective Study of Pravastatin in the Elderly at Risk (PROSPER). A detailed description of the study has been published elsewhere. PROSPER was a prospective multicenter randomized placebo-controlled trial to assess whether treatment with pravastatin diminishes the risk of major vascular events in elderly. Between December 1997 and May 1999, we screened and enrolled subjects in Scotland (Glasgow), Ireland (Cork), and the Netherlands (Leiden). Men and women aged 70-82 years were recruited if they had pre-existing vascular disease or increased risk of such disease because of smoking, hypertension, or diabetes. A total number of 5,804 subjects were randomly assigned to pravastatin or placebo. A large number of prospective tests were performed including Biobank tests and cognitive function measurements. A whole genome wide screening has been performed in the sequential PHASE project. Of 5,763 subjects DNA was available for genotyping. Genotyping was performed with the Illumina 660K beadchip, after QC (call rate <95%) 5,244 subjects and 557,192 SNPs were left for analysis. These SNPs were imputed to 2.5 million SNPs based on the HAPMAP built 36 with MACH imputation software. The study was approved by the institutional ethics review boards of centers of Cork University (Ireland), Glasgow University (Scotland) and Leiden University Medical Center (the Netherlands) and all participants gave written informed consent.

RS - Rotterdam Study
Rotterdam Study, a prospective population-based cohort study. Details regarding design, objectives, and methods of the Rotterdam Study have been described in detail. (Hofman et al, 2016) In short, the Rotterdam study started in 1989 with an initial cohort of 7,983 persons (out of 10,215 invitees; response rate 78%) 55 years of age or older living in the Ommoord district in the city of Rotterdam in the Netherlands. In 2000, 3,011 participants (out of 4,472 invitees, response rate 67%) who had become 55 years of age or moved into the study district were added to the cohort. As of In 2006, a further extension of the cohort was initiated in which 3932 subjects were included, aged 45–54 years,

out of 6057 invited, living in the Ommoord district. In summer of 2016, the recruitment of another extension started that targeted participants aged 40 years and over. The establishment of this extension is expected to be completed by early 2020 and to yield around 3000 new participants. The participants were all extensively examined at study entry (i.e. baseline) and subsequent follow-up visits that take place every 3 to 6 years. They were interviewed at home (2 h) and then underwent an extensive set of examinations (a total of 5 h) in a specially built research facility in the centre of the district. These examinations focused on possible causes of invalidating diseases in the elderly in a clinically state-of-the-art manner, as far as the circumstances allowed. The emphasis was put on imaging (of heart, blood vessels, eyes, skeleton and later brain) and on collecting biospecimens that enabled further in-depth molecular and genetic analyses. Approximately every 4-5 years follow-up examinations are conducted. Examinations consist of a home interview and an extensive set of test at a research facility in the study district. By linking the general practitioners' and municipality records to the study database, participants are continuously monitored for major morbidity and mortality.

SardiNIA - SardiNIA Project
To identify genetic bases for prominent age-associated changes, including cardiovascular risk factors and determinants of personality traits, in a founder population. The results of the study will extend the studies of aging-associated conditions of outbred populations.

SHIP-START and SHIP-TREND - Study of Health in Pomerania
The Study of Health In Pomerania is a prospective longitudinal population-based cohort study in Western Pomerania assessing the prevalence and incidence of common diseases and their risk factors. SHIP encompasses the two independent cohorts SHIP-START and SHIP-TREND. The detailed study design has been published previously. In brief, participants aged 20 to 79 with German citizenship and principal residency in the study area were recruited from a random sample of residents living in the three local cities (with 17,076 to 65,977 inhabitants), 12 towns (with 1,516 to 3,044 inhabitants) as well as 17 out of 97 (with less than 1,500 inhabitants) randomly selected smaller towns. Individuals were randomly selected in proportion to the population size of the community and stratified by age and sex. For SHIP-START, a total of 4,308 participants were recruited between 1997 and 2001. Between 2008 and 2012 a total of 4,420 participants were recruited in the SHIP-TREND cohort. Individuals were invited to the SHIP study center for computer-assisted personal interviews and extensive physical examinations. Individuals of both cohorts were analyzed separately.

TWINSUK - TWINSUK
There are currently >13,500 twins registered participants in the TwinsUK study, of which over 9,000 are actively participating. The twins are aged 16 to 100 with approximately equal numbers of identical (MZ) and non-identical (DZ) twins and are predominantly female (80%) for historical reasons. Clinical, physiological, behavioral and lifestyle data is collected at either twin visits or via self-administered questionnaires, which volunteers complete either once or twice a year via the post or email. All studies have ethical approval from the Guy's and St Thomas' (GSTT) Ethics Committee.

UK Biobank - UK Biobank Study
UK Biobank (UKB, www.ukbiobank.ac.uk) is a large longitudinal biobank study in the United Kingdom which was established to improve understanding of the genetic and environmental causes of common diseases including CVDs. In addition to self-reported disease outcomes and extensive health and life-style questionnaire data, UKB participants are being tracked through their NHS records and national registries (including cause of death and Hospital Episode Statistics). In 2017, UKB released the genotypes of 488,377 participants profiled with a custom SNP array. Genotyping QC was performed centrally by UKB, and genotypes imputed to Haplotype Reference Consortium (HRC) panel were released for 488,377 participants. For the UKB-EXECG sub-cohort, ECG measures were calculated from 4-lead ECGs (CAM-USB 6.5, Cardiosoft v6.51) recorded during a 15 second rest period prior to an exercise test while subjects were sitting on a stationary bike (eBike, Firmware v1.7). Electrodes were placed on the right and left antecubital fossae, and left and right wrist and the ECG was sampled at 500 Hz; lead I was used to derive ECG measures. To reduce the influence of noise, QT, JT and QRS were measured from a signal averaged ECG waveform computed from the

heartbeats available in the 15s trace. For this, we first identified the QRS-complexes using fully automatic in-house algorithms. Before averaging, we removed ectopic beats and artefacts, as well as beats with an RR interval longer or shorter than 10ms compared to the mean RR interval were not included for averaging. A signal-averaged heartbeat was then computed from the remaining beats provided that the number of available beats was not less than 5. For the UKB-12lead sub-cohort, ECG measures were calculated from a resting 12-lead ECG. As described above, to reduce the influence of noise, ECG measures were calculated from signal averaged ECG wave forms computed from lead I. When individuals had both 12-lead ECG and exercise test ECG data, the 12-lead recording was used, to ensure no overlap between sub-cohorts.

VIKING - Viking Health Study
The Viking Health Study - Shetland (VIKING) is a family-based, cross-sectional study that seeks to identify genetic factors influencing cardiovascular and other disease risk in the population isolate of the Shetland Isles in northern Scotland. Genetic diversity in this population is decreased compared to Mainland Scotland, consistent with the high levels of endogamy historically. 2105 participants were recruited between 2013 and 2015, most having at least three grandparents from Shetland. Fasting blood samples were collected and many health-related phenotypes and environmental exposures were measured in each individual. All participants gave informed consent and the study was approved by the South East Scotland Research Ethics Committee.

WHI - Women's Health Initiative
The Women's Health Initiative (WHI) is a long-term national health study focused on strategies for preventing heart disease, breast and colorectal cancer, and osteoporotic fractures in postmenopausal women. Launched in 1993, the WHI enrolled 161,808 women aged 50-79 into one or more randomized Clinical Trials (CT), testing the health effects of hormone therapy (HT), dietary modification (DM), and/or calcium and Vitamin D supplementation (CaD) or to an Observational Study (OS).
This ground-breaking study changed the way health care providers prevent and treat some of the major diseases impacting postmenopausal women. Results from the WHI Hormone Trials have been estimated to have already saved $35.2 billion in direct medical costs in the US alone. To date, WHI has published over 1,400 articles and approved and funded 289 ancillary studies. The GWAS data used in this paper comes from six ancillary studies.

YFS - Young Finns Study
The YFS is a population-based follow up-study started in 1980. The main aim of the YFS is to determine the contribution made by childhood lifestyle, biological and psychological measures to the risk of cardiovascular diseases in adulthood. In 1980, over 3,500 children and adolescents all around Finland participated in the baseline study. The follow-up studies have been conducted mainly with 3-year intervals. The 27-year follow-up study was conducted in 2007 (ages 30-45 years) with 2,204 participants. The study was approved by the local ethics committees (University Hospitals of Helsinki, Turku, Tampere, Kuopio and Oulu) and was conducted following the guidelines of the Declaration of Helsinki. All participants gave their written informed consent.

Supplementary Note 2 – Heritability estimates and comparison with previous studies

The SNP-based heritability estimates calculated in this study for QT are similar to previously reported values (30-35%). Heritability estimates for JT have not previously been reported in the literature. However, as anticipated due to their high genetic correlation, we obtained similar estimates to QT. Our estimates for QRS are lower than previously reported (23-33%); however, previous calculations were performed on substantially smaller sample sizes resulting in wide confidence intervals. The percentage variance of QT, JT and QRS explained by our findings suggests further studies with larger sample sizes, including individuals with whole genome sequencing data, will likely yield additional loci. However, the effect sizes of additional common variants are likely to be progressively smaller than identified in this study. Larger studies of rare variants may therefore identify a greater proportion of the unexplained heritability, as may gene x environment interaction studies.

Supplementary Note 3 – Study Acknowledgements

ARIC - Atherosclerosis Risk in Communities
The authors thank the staff and participants of the ARIC study for their important contributions.

BioMe - The IPM Bio*Me* Biobank
The Mount Sinai BioMe Biobank is supported by The Andrea and Charles Bronfman Philanthropies.

BRIGHT - British Genetics of Hypertension
The BRIGHT study is extremely grateful to all the patients who participated in the study and the BRIGHT nursing team. This work forms part of the research program of the National Institutes of Health Research (NIHR Cardiovascular Biomedical Research) Cardiovascular Biomedical Unit at Barts and The London, QMUL. P.B.M. wishes to acknowledge the NIHR Cardiovascular Biomedical Research Unit at Barts and The London, Queen Mary University of London, UK for support.

CHRIS - The Cooperative Health Research in South Tyrol study
The CHRIS study is a collaborative effort between the Center for Biomedicine of the European Academy of Bolzano/Bozen (EURAC) and the Healthcare System of the Autonomous Province of Bolzano (Südtiroler Sanitätsbetrieb/Azienda Sanitaria dell'Alto Adige). The CHRIS Study is affiliated to the "German National Cohort" (Germany) and is indebted with the investigators of this study for their support in the study protocol definition. Full acknowledgements for the CHRIS study are reported here: http://translational-medicine.biomedcentral.com/articles/10.1186/s12967-015-0704-9#Declarations.

ERF - Erasmus Rucphen Family Study
We are grateful to all study participants and their relatives, general practitioners and neurologists for their contributions to the ERF study and to P Veraart for her help in genealogy, J Vergeer for the supervision of the laboratory work and P Snijders for his help in data collection.

FINCAVAS - Finnish Cardiovascular Study
The authors thank the staff of the Department of Clinical Physiology for collecting the exercise test data.

GAPP - Genetic and phenotypic determinants of blood pressure and other cardiovascular risk factors
We thank the GAPP staff and all GAPP study participants for their important contributions

GS20 - Generation Scotland: Scottish Family Health Study
We are grateful to all the families who took part, the general practitioners and the Scottish School of Primary Care for their help in recruiting them, and the whole Generation Scotland team, which includes interviewers, computer and laboratory technicians, clerical workers, research scientists, volunteers, managers, receptionists, healthcare assistants and nurses.

HCHS/SOL - Hispanic Community Health Survey/Study of Latinos
Hispanic Community Health Study/Study of Latinos (HCHS/SOL): We thank the participants and staff of the HCHS/SOL study for their contributions to this study. The baseline examination of HCHS/SOL was carried out as a collaborative study supported by contracts from the National Heart, Lung, and Blood Institute (NHLBI) to the University of North Carolina (N01-HC65233), University of Miami (N01-HC65234), Albert Einstein College of Medicine (N01-HC65235), Northwestern University (N01-HC65236) and San Diego State University (N01-HC65237).

Health ABC - Health, Aging, and Body Composition Study
This research was supported by NIA contracts N01AG62101, N01AG62103, and N01AG62106. The genome-wide association study was funded by NIA grant 1R01AG032098-01A1 to Wake Forest University Health Sciences and genotyping services were provided by the Center for Inherited

Disease Research (CIDR). CIDR is fully funded through a federal contract from the National Institutes of Health to The Johns Hopkins University, contract number HHSN268200782096C. This research was supported in part by the Intramural Research Program of the NIH, National Institute on Aging.

INGI-CAR - INGI-CARLANTINO / INGI-FVG - INGI- Friuli Venezia Giulia
We are very grateful to the municipal administrators for their collaboration on the project and for logistic support. We would like to thank all participants to this study.

INTER99 - Inter99
The Inter99 was initiated by Torben Jørgensen (PI), Knut Borch-Johnsen (co-PI), Hans Ibsen and Troels F. Thomsen. The steering committee comprises the former two and Charlotta Pisinger.

JHS - Jackson Heart Study
We thank the Jackson Heart Study (JHS) participants and staff for their contributions to this work.

KORA F3/S4 - Kooperative Gesundheitsforschung in der Region Augsburg F3/S4
The KORA study was initiated and financed by the Helmholtz Zentrum München – German Research Center for Environmental Health, which is funded by the German Federal Ministry of Education and Research (BMBF) and by the State of Bavaria. Furthermore, KORA research was supported within the Munich Center of Health Sciences (MC-Health), Ludwig-Maximilians-Universität, as part of LMUinnovativ.

LIFELINES - LifeLines, a three-generation cohort study and biobank
We thank U. Bultmann (1), J.M. Geleijnse (2), P. van der Harst (3), S. Mulder (4), J.G.M. Rosmalen (5), E.F.C. van Rossum (6), H.A. Smit (7), M. Swertz (8), E.A.L.M. Verhagen (9).
(1) Department of Social Medicine, University Medical Center Groningen, University of Groningen, The Netherlands (2) Department of Human Nutrition, Wageningen University, The Netherlands (3) Department of Cardiology, University Medical Center Groningen, University of Groningen, The Netherlands (4) Lifelines Cohort Study, The Netherlands (5) Interdisciplinary Center of Psychopathology of Emotion Regulation (ICPE), Department of Psychiatry, University Medical Center Groningen, University of Groningen, The Netherlands (6) Department of Endocrinology, Erasmus Medical Center, Rotterdam, The Netherlands (7) Department of Public Health, University Medical Center Utrecht, The Netherlands (8) Department of Genetics, University Medical Center Groningen, University of Groningen, The Netherlands (9) Department of Public and Occupational Health, VU Medical Center, Amsterdam, The Netherlands

NEO - Netherlands Epidemiology of Obesity
The authors of the NEO study thank all individuals who participated in the Netherlands Epidemiology in Obesity study, all participating general practitioners for inviting eligible participants and all research nurses for collection of the data. We thank the NEO study group, Pat van Beelen, Petra Noordijk and Ingeborg de Jonge for the coordination, lab and data management of the NEO study. We also thank Arie Maan for the analyses of the electrocardiograms.

OOA - Old Order Amish
We gratefully thank our Amish community and research volunteers for their long-standing partnership in research, and acknowledge the dedication of our Amish liaisons, field workers and the Amish Research Clinic staff, without which these studies would not have been possible.

ORCADES - Orkney Complex Disease Study
DNA extractions were performed at the Wellcome Trust Clinical Research Facility in Edinburgh. We would like to acknowledge the invaluable contributions of the research nurses in Orkney, the administrative team in Edinburgh and the people of Orkney.

PIVUS - Prospective Investigation of the Vasculature of Uppsala Seniors
DNA extractions were performed at the Wellcome Trust Clinical Research Facility in Edinburgh. We would like to acknowledge the invaluable contributions of the research nurses in Orkney, the administrative team in Edinburgh and the people of Orkney.

PROSPER - PROSpective study of pravastatin in the elderly at Risk for vascular disease
The PROSPER study was supported by an investigator initiated grant obtained from Bristol-Myers Squibb. J.Wouter Jukema is an Established Clinical Investigator of the Netherlands Heart Foundation (grant 2001 D 032). Support for genotyping was provided by the seventh framework program of the European commission (grant 223004) and by the Netherlands Genomics Initiative (Netherlands Consortium for Healthy Aging grant 050-060-810).

RS - Rotterdam Study
We thank Pascal Arp, Mila Jhamai, Dr Michael Moorhouse, Marijn Verkerk, and Sander Bervoets for their help in creating the GWAS database. The authors are very grateful to the participants and staff from the Rotterdam Study, the participating general practitioners and the pharmacists.

SardiNIA - SardiNIA Project
We thank the many volunteers who generously participated in this study, the Mayors and citizens of the Sardinian towns involved, the head of the Public Health Unit ASL4, and the province of Ogliastra for their volunteerism and cooperation. In addition, we are grateful to the Mayor and the administration in Lanusei for providing and furnishing the clinic site. We are grateful to the physicians Angelo Scuteri, Marco Orrù, Maria Grazia Pilia, Liana Ferreli, Francesco Loi, nurses Paola Loi, Monica Lai and Anna Cau who carried out participant physical exams; the recruitment personnel Susanna Murino; and Mariano Dei, Sandra Lai, Andrea Maschio, Fabio Busonero for genotyping.

SHIP and SHIP-TREND - Study of Health in Pomerania
SHIP is part of the Community Medicine Research Network of the University Medicine Greifswald, which is supported by the German Federal State of Mecklenburg- West Pomerania.

UK Biobank - UK Biobank Study
This research has been conducted using the UK Biobank Resource (application 8256 - Understanding genetic influences in the response of the cardiac electrical system to exercise). This work forms part of the research program of the National Institutes of Health Research (NIHR Cardiovascular Biomedical Research) Cardiovascular Biomedical Centre at Barts and The London, QMUL. PD Lambiase acknowledges support from the UCLH Biomedicine NIHR. JR acknowledges support from the People Programme (Marie Curie Actions) of the European Union's Seventh Framework Programme (FP7/2007/2013) under REA grant agreement 608765

VIKING - Viking Health Study
DNA extractions and genotyping were performed at the Edinburgh Clinical Research Facility, University of Edinburgh. We would like to acknowledge the invaluable contributions of the research nurses in Shetland, the administrative team in Edinburgh and the people of Shetland.

WHI - Women's Health Initiative
The WHI program is funded by the National Heart, Lung, and Blood Institute, National Institutes of Health. The authors thank the WHI investigators and staff for their dedication, and the study participants for making the program possible. A full listing of WHI investigators can be found at: https://www-whi-org.s3.us-west-2.amazonaws.com/wp-content/uploads/WHI-Investigator-Long-List.pdf.

YFS - Young Finns Study
We thank the teams that collected data at all measurement time points; the persons who participated as both children and adults in these longitudinal studies; and biostatisticians Irina Lisinen, Johanna

Ikonen, Noora Kartiosuo, Ville Aalto, and Jarno Kankaanranta for data management and statistical advice.

Additional acknowledgements
This research utilized Queen Mary's Apocrita HPC facility, supported by QMUL Research-IT. http://doi.org/10.5281/zenodo.438045

Supplementary Note 4 – Study Funding

ARIC - Atherosclerosis Risk in Communities
The Atherosclerosis Risk in Communities study has been funded in whole or in part with Federal funds from the National Heart, Lung, and Blood Institute, National Institutes of Health, Department of Health and Human Services, under Contract nos. (HHSN268201700001I, HHSN268201700002I, HHSN268201700003I, HHSN268201700004I, HHSN268201700005I). Funding was also supported by R01HL087641, R01HL059367 and R01HL086694; National Human Genome Research Institute contract U01HG004402; and National Institutes of Health contract HHSN268200625226C. Infrastructure was partly supported by Grant Number UL1RR025005, a component of the National Institutes of Health and NIH Roadmap for Medical Research.

Bambui - Brazilian Bambuí Cohort Study of Ageing
Brazilian Ministry of Health (DECIT/MS, EPIGEN-Brazil Project), Brazilian Ministry of Science and Technology (Financiadora de Estudos e Projetos (FINEP), Brazilian Conselho Nacional de Desenvolvimento Científico e Tecnologico (CNPq), Fundação de Amparo a Pesquisa do Estado de Minas Gerais (FAPEMIG).

BRIGHT - British Genetics of Hypertension
This work was supported by the Medical Research Council (MRC) of Great Britain (grant number G9521010D) and the British Heart Foundation (grant number PG/02/128).

CHRIS - The Cooperative Health Research in South Tyrol study
The CHRIS study was funded by the Department of Innovation, Research, and University of the Autonomous Province of Bolzano-South Tyrol.

CHS - Cardiovascular Health Study
Cardiovascular Health Study: This CHS research was supported by NHLBI contracts HHSN268200960009C, HHSN268201200036C, HHSN268200800007C, HHSN268201800001C, N01HC55222, N01HC85079, N01HC85080, N01HC85081, N01HC85082, N01HC85083, N01HC85086, 75N92021D00006; and NHLBI grants U01HL080295, R01HL085251, R01HL087652, R01HL105756, R01HL103612, R01HL120393, and U01HL130114 with additional contribution from the National Institute of Neurological Disorders and Stroke (NINDS). Additional support was provided through R01AG023629 from the National Institute on Aging (NIA). A full list of principal CHS investigators and institutions can be found at CHS-NHLBI.org. The provision of genotyping data was supported in part by the National Center for Advancing Translational Sciences, CTSI grant UL1TR001881, and the National Institute of Diabetes and Digestive and Kidney Disease Diabetes Research Center (DRC) grant DK063491 to the Southern California Diabetes Endocrinology Research Center. The content is solely the responsibility of the authors and does not necessarily represent the official views of the National Institutes of Health.

ERF - Erasmus Rucphen Family Study
The ERF study as a part of EUROSPAN (European Special Populations Research Network) was supported by European Commission FP6 STRP grant number 018947 (LSHG-CT-2006-01947) and also received funding from the European Community's Seventh Framework Programme (FP7/2007-2013)/grant agreement HEALTH-F4-2007-201413 by the European Commission under the programme “Quality of Life and Management of the Living Resources” of 5th Framework Programme (no. QLG2-CT-2002-01254). The ERF study was further supported by ENGAGE consortium and CMSB. High-throughput analysis of the ERF data was supported by joint grant from Netherlands Organisation for Scientific Research and the Russian Foundation for Basic Research (NWO-RFBR 047.017.043).

FINCAVAS - Finnish Cardiovascular Study
The Finnish Cardiovascular Study (FINCAVAS) has been financially supported by the Competitive Research Funding of the Tampere University Hospital (Grant 9M048 and 9N035), the Finnish

Cultural Foundation, the Finnish Foundation for Cardiovascular Research, the Emil Aaltonen Foundation, Finland, the Tampere Tuberculosis Foundation, EU Horizon 2020 (grant 755320 for TAXINOMISIS and grant 848146 for To Aition), and the Academy of Finland grant 322098.

FinGesture – Finnish Genetic Study for Arrhythmic Events
This work was supported by the Juselius Foundation (Helsinki, Finland); the Council of Health of the Academy of Finland (Helsinki, Finland); the Montreal Heart Institute Foundation; Finnish Foundation for Cardiovascular Research (Helsinki, Finland); and Erkko Foundation (Helsinki, Finland).

GAPP - Genetic and phenotypic determinants of blood pressure and other cardiovascular risk factors
The GAPP study was supported by the Liechtenstein Government, the Swiss Heart Foundation, the Swiss Society of Hypertension, the University of Basel, the University Hospital Basel, the Hanela Foundation, the Mach-Gaensslen Foundation, Schiller AG, and Novartis.

GS20 - Generation Scotland: Scottish Family Health Study
Generation Scotland received core support from the Chief Scientist Office of the Scottish Government Health Directorates [CZD/16/6] and the Scottish Funding Council [HR03006]. Genotyping of the GS:SFHS samples was carried out by the Genetics Core Laboratory at the t Clinical Research Facility, University of Edinburgh, Scotland and was funded by the Medical Research Council UK and the Wellcome Trust (Wellcome Trust Strategic Award “STratifying Resilience and Depression Longitudinally” (STRADL) Reference 104036/Z/14/Z).

HCHS/SOL - Hispanic Community Health Survey/Study of Latinos
This work was supported by funding from the National Heart, Lunch and Blood Institute (N01-HC65233, N01-HC65234, N01-HC65235, N01-HC65236, N01-HC65237, and T32HL7055).

Health ABC - Health, Aging, and Body Composition Study
This research was supported by NIA contracts N01AG62101, N01AG62103, and N01AG62106. The genome-wide association study was funded by NIA grant 1R01AG032098-01A1 to Wake Forest University Health Sciences and genotyping services were provided by the Center for Inherited Disease Research (CIDR). CIDR is fully funded through a federal contract from the National Institutes of Health to The Johns Hopkins University, contract number HHSN268200782096C. This research was supported in part by the Intramural Research Program of the NIH, National Institute on Aging.

INGI-CAR - INGI-CARLANTINO / INGI-FVG - INGI-Friuli Venezia Giulia
Italian Ministry of Health - RC 35/17; Italian Ministry of Education, University and Research, D70-RESRICGIROTTO to GG

INTER99 - Inter99
The study was financially supported by research grants from the Danish Research Council, the Danish Centre for Health Technology Assessment, Novo Nordisk Inc., Research Foundation of Copenhagen County, Ministry of Internal Affairs and Health, the Danish Heart Foundation, the Danish Pharmaceutical Association, the Augustinus Foundation, the Ib Henriksen Foundation, the Becket Foundation, and the Danish Diabetes Association.

JHS - Jackson Heart Study
The JHS is supported by contracts HHSN268201800010, HHSN268201800011, HHSN268201800012, HHSN268201800013, HHSN268201800014, and HHSN268201800015 from the National Heart, Lung, and Blood Institute and the National Institute on Minority Health and Health Disparities.

KORA F3/S4 - Kooperative Gesundheitsforschung in der Region Augsburg F3/S4

The KORA study was initiated and financed by the Helmholtz Zentrum München – German Research Center for Environmental Health, which is funded by the German Federal Ministry of Education and Research (BMBF) and by the State of Bavaria. Furthermore, KORA research was supported within the Munich Center of Health Sciences (MC-Health), Ludwig-Maximilians-Universität, as part of LMUinnovativ.

LIFELINES - LifeLines, a three-generation cohort study and biobank
The LifeLines Cohort Study, and generation and management of GWAS genotype data for the LifeLines Cohort Study is supported by the Netherlands Organization of Scientific Research NWO (grant 175.010.2007.006), the Economic Structure Enhancing Fund (FES) of the Dutch government, the Ministry of Economic Affairs, the Ministry of Education, Culture and Science, the Ministry for Health, Welfare and Sports, the Northern Netherlands Collaboration of Provinces (SNN), the Province of Groningen, University Medical Center Groningen, the University of Groningen, Dutch Kidney Foundation and Dutch Diabetes Research Foundation..

MESA - Multi-Ethnic Study of Atherosclerosis
MESA and the MESA SHARe projects are conducted and supported by the National Heart, Lung, and Blood Institute (NHLBI) in collaboration with MESA investigators. Support for MESA is provided by contracts 75N92020D00001, HHSN268201500003I, N01-HC-95159, 75N92020D00005, N01-HC-95160, 75N92020D00002, N01-HC-95161, 75N92020D00003, N01-HC-95162, 75N92020D00006, N01-HC-95163, 75N92020D00004, N01-HC-95164, 75N92020D00007, N01-HC-95165, N01-HC-95166, N01-HC-95167, N01-HC-95168, N01-HC-95169, UL1-TR-000040, UL1-TR-001079, and UL1-TR-001420, UL1TR001881, and DK063491. Funding for SHARe genotyping was provided by NHLBI Contract N02-HL-64278. Genotyping was performed at Affymetrix (Santa Clara, California, USA) and the Broad Institute of Harvard and MIT (Boston, Massachusetts, USA) using the Affymetrix Genome-Wide Human SNP Array 6.0. The authors thank the other investigators, the staff, and the participants of the MESA study for their valuable contributions. A fill list of participating MESA investigators and institutes can be found at http://www.mesa-nhlbi.org.

NEO - Netherlands Epidemiology of Obesity
The genotyping in the NEO study was supported by the Centre National de Génotypage (Paris, France), headed by Jean-Francois Deleuze. The NEO study is supported by the participating Departments, the Division and the Board of Directors of the Leiden University Medical Center, and by the Leiden University, Research Profile Area Vascular and Regenerative Medicine.

NFBC1966 – Northern Finland Birth Cohort of 1966
The NFBC1966 Study is conducted and supported by the National Heart, Lung, and Blood Institute (NHLBI [grant number: 5R01HL087679-02] through the STAMPEED program [genotyping, grant number: 1RL1MH083268-01]), in collaboration with the Broad Institute, UCLA, University of Oulu, and the National Institute for Health and Welfare in Finland. The NFBC1966 has received core funding for data generation and curation from the Academy of Finland (project grants 104781, 120315, 129269, 1114194, 24300796, 85547, 285547 (EGEA)), University Hospital Oulu, Finland (75617), ERDF European Regional Development Fund Grant no. 539/2010 A31592 and the EU H2020--PHC-2014 DynaHEALTH action (grant no. 633595).

OOA - Old Order Amish
The Amish studies are supported by grants and contracts from the NIH, including R01 AG18728, R01 HL088119, U01 GM074518, U01 HL072515, U01 HL84756, U01 HL137181, R01 DK54261,the University of Maryland General Clinical Research Center, grant M01 RR 16500, and the Mid-Atlantic Nutrition Obesity Research Center grant P30 DK72488, the Baltimore Diabetes Research and Training Center grant P60DK79637.

ORCADES - Orkney Complex Disease Study
The Orkney Complex Disease Study (ORCADES) was supported by the Chief Scientist Office of the Scottish Government (CZB/4/276, CZB/4/710), a Royal Society URF to J.F.W., the MRC Human

Genetics Unit quinquennial programme "QTL in Health and Disease", Arthritis Research UK and the European Union framework program 6 EUROSPAN project (contract no. LSHG-CT-2006-018947).

PIVUS - Prospective Investigation of the Vasculature of Uppsala Seniors
PIVUS was supported by Knut and Alice Wallenberg Foundation (Wallenberg Academy Fellow), European Research Council (ERC Starting Grant), Swedish Diabetes Foundation (2013-024), Swedish Research Council (2012-1397, 2012-1727, and 2012-2215), Marianne and Marcus Wallenberg Foundation, County Council of Dalarna, Dalarna University, and Swedish Heart-Lung Foundation (20120197). Genotyping was funded by the Wellcome Trust under awards WT064890 and WT086596. Analysis of genetic data was funded by the Wellcome Trust under awards WT098017 and WT090532.

PREVEND - Prevention of REnal and Vascular ENd stage Disease
PREVEND genetics is supported by the Dutch Kidney Foundation (Grant E033), the EU project grant GENECURE (FP-6 LSHM CT 2006 037697), the National Institutes of Health (grant 2R01LM010098), The Netherlands organization for health research and development (NWO-Groot grant 175.010.2007.006, NWO VENI grant 916.761.70, ZonMw grant 90.700.441), and the Dutch Inter University Cardiology Institute Netherlands (ICIN).

PROSPER - PROSpective study of pravastatin in the elderly at Risk for vascular disease
The PROSPER study was supported by an investigator initiated grant obtained from Bristol-Myers Squibb. Support for genotyping was provided by the seventh framework program of the European commission (grant 223004) and by the Netherlands Genomics Initiative (Netherlands Consortium for Healthy Aging grant 050-060-810).

RS - Rotterdam Study
The Rotterdam Study is supported by the Erasmus Medical Center and Erasmus University Rotterdam; the Netherlands Organization for Scientific Research (NWO); the Netherlands Organization for Health Research and Development (ZonMw); the Research Institute for Diseases in the Elderly (RIDE); the Netherlands Heart Foundation; the Ministry of Education, Culture and Science; the Ministry of Health Welfare and Sports; the European Commission; and the Municipality of Rotterdam. Support for genotyping was provided by the Netherlands Organisation of Scientific Research NWO Investments (nr. 175.010.2005.011, 911-03-012), the Research Institute for Diseases in the Elderly (014-93-015; RIDE2), the Netherlands Genomics Initiative (NGI)/Netherlands Consortium for Healthy Aging (NCHA) project nr. 050- 060-810. The GWA study was funded by the Netherlands Organisation of Scientific Research NWO Investments (nr. 175.010.2005.011, 911-03-012), the Research Institute for Diseases in the Elderly (014-93-015; RIDE2), the Netherlands Genomics Initiative (NGI)/Netherlands Consortium for Healthy Aging (NCHA) project nr. 050-060-810.

SardiNIA - SardiNIA Project
This study was funded in part by the National Institutes of Health (National Institute on Aging, National Heart Lung and Blood Institute, and National Human Genome Research Institute). This research was supported by the Intramural Research Program of the NIH, National Institute on Aging, with contracts N01-AG-1-2109 and HHSN271201100005C and by Sardinian Autonomous Region (L.R. no. 7/2009) grant cRP3-154.

SHIP-START and SHIP-TREND - Study of Health in Pomerania
SHIP is supported by the German Federal Ministry of Education and Research (Bundesministerium für Bildung und Forschung (BMBF); grants 01ZZ9603, 01ZZ0103, and 01ZZ0403) and the German Research Foundation (Deutsche Forschungsgemeinschaft (DFG); grant GR 1912/5-1). SHIP-START and SHIP-TREND are part of the Community Medicine Research net (CMR) of the University of Greifswald which is funded by the BMBF as well as the Ministry for Education, Science and Culture and the Ministry of Labor, Equal Opportunities, and Social Affairs of the Federal State of Mecklenburg-West Pomerania. The CMR encompasses several research projects that share data from

SHIP. Genome-wide data have been supported by a joint grant from Siemens Healthcare, Erlangen, Germany and the Federal State of Mecklenburg-West Pomerania.

TWINSUK - TWINSUK

TwinsUK receives funding from the Wellcome Trust (212904/Z/18/Z), Medical Research Council (AIMHY; MR/M016560/1) and European Union (H2020 contract #733100). TwinsUK is supported by the National Institute for Health Research (NIHR)-funded BioResource, Clinical Research Facility and Biomedical Research Centre based at Guy's and St Thomas' NHS Foundation Trust in partnership with King's College London. Support is also acknowledged from the British Heart Foundation (grant number PG/12/38/29615).

VIKING - Viking Health Study

The Viking Health Study – Shetland (VIKING) was supported by the MRC Human Genetics Unit quinquennial programme grant "QTL in Health and Disease". Linda Repetto is supported by a University of Edinburgh studentship.

WHI - Women's Health Initiative

The WHI program is funded by the National Heart, Lung, and Blood Institute, National Institutes of Health, U.S. Department of Health and Human Services through contracts HHSN268201600018C, HHSN268201600001C, HHSN268201600002C, HHSN268201600003C, and HHSN268201600004C. Scientific Computing Infrastructure at the Fred Hutchinson Cancer Center is funded by ORIP grant S10OD028685. The Modification of PM-Mediated Arrhythmogenesis in Populations (WHI-MOPMAP) was funded by the NIEHS (R01-ES017794, Whitsel).

YFS - Young Finns Study

The Young Finns Study has been financially supported by the Academy of Finland: grants 322098, 286284, 134309 (Eye), 126925, 121584, 124282, 129378 (Salve), 117787 (Gendi), and 41071 (Skidi); the Social Insurance Institution of Finland; Competitive State Research Financing of the Expert Responsibility area of Kuopio, Tampere and Turku University Hospitals (grant X51001); Juho Vainio Foundation; Paavo Nurmi Foundation; Finnish Foundation for Cardiovascular Research ; Finnish Cultural Foundation; The Sigrid Juselius Foundation; Tampere Tuberculosis Foundation; Emil Aaltonen Foundation; Yrjö Jahnsson Foundation; Signe and Ane Gyllenberg Foundation; Diabetes Research Foundation of Finnish Diabetes Association; EU Horizon 2020 (grant 755320 for TAXINOMISIS and grant 848146 for To Aition); European Research Council (grant 742927 for MULTIEPIGEN project); Tampere University Hospital Supporting Foundation and Finnish Society of Clinical Chemistry.
