# Supplementary material for: Genetic analyses of the electrocardiographic QT interval and its components identify additional loci and pathways
Source: Nat Commun. 2022 Sep 1;13:5144. doi: 10.1038/s41467-022-32821-z (PMC9436946; doi:10.1038/s41467-022-32821-z)
Supplement: Supplementary file 3 — Description of Additional Supplementary Files [file 41467_2022_32821_MOESM3_ESM.pdf]

Description of Additional Supplementary Files

File Name: Supplementary Data 1
Description: **Study information for all participating studies.** EA; European ancestry, AA; African ancestry, HA; Hispanic/Latino, EAS; East Asian ancestry, SAS; South Asian Ancestry

File Name: Supplementary Data 2
Description: **Per study (and sub-study) summary of genotyping and GWAS software information.** EA; European ancestry, AA; African ancestry, HA; Hispanic/Latino, EAS; East Asian ancestry, SAS; South Asian Ancestry

File Name: Supplementary Data 3
Description: **Per study summary statistics for ECG measures and covariates.** EA; European ancestry, AA; African ancestry, HA; Hispanic/Latino, EAS; East Asian ancestry, SAS; South Asian Ancestry. ECG data for the UK Biobank was derived from two sources: Resting component of the exercise bike test (EXECG) and a resting 12-lead ECG (12-lead) and were therefore analysed separately to take into account the different modes of data acquisition, 12-lead resting ECG measures were used when individuals had both tests performed.

File Name: Supplementary Data 4
Description: **Previously reported variants from GWAS for resting QT, JT and QRS.** Chr, chromosome; Position, base pair position in build 37; P-value, P-value of association in the reporting study; PMID, Pubmed ID; Significant locus in our respective multi-ancestry meta-analysis, Whether the locus was significant in our multi-ancestry meta-analysis (Y = Yes, N = No). When a locus was not a significant locus in our study, a look up was performed of the previously reported variant(s) and the lowest P-value reported; *QT association from a reported sex-stratified analysis

File Name: Supplementary Data 5
Description: **Lead variants from independent loci for QT GWAS meta-analysis.** Summary statistics shown for lead variants at independent loci for each genome-wide association study meta-analysis. Study level linear regression summary statistics (two-sided) were meta-analysed using METAL, where effect size estimates were weighted using the inverse of the corresponding standard errors. A Bonferroni-corrected threshold for multiple testing ($P<5\times10^{-8}$) was used to declare genome-wide significance. CHRPOSID, Unique variant identifier; Chr, chromosome; Position, base pair position in build 37; EA, Effect allele; OA, Other allele; EAF, Effect allele frequency; Beta, Effect estimate from linear regression (inverse normal transformed phenotype); SE, Standard error; P-value, Beta (ms), corresponding effect size estimate from untransformed meta-analysis; Het I2, Heterogeneity I2 statistic; P-value of association; *result from X-chromosome male stratified analysis, †locus significant in ancestry-specific analysis but not in multi-ancestry meta-analysis.

File Name: Supplementary Data 6
Description: **Conditional analysis results for QT, JT and QRS traits in individuals of European ancestry.** Findings from joint and conditional analyses with Genome-wide Complex Trait Analysis (GCTA). Type, 'lead' for the most significant variant in the European meta-analysis at the locus; 'conditional' for identified independent variants identified in UK Biobank. Chr, chromosome;

CHRPOSID, Unique variant identifier, Position, base pair position in build 37; EA, effect allele; EAF, effect allele frequency; Beta, effect estimate from linear regression (inverse normal transformed phenotype); SE, standard error; P-value, P-value of association from the GWAS meta-analysis; Beta Joint, beta estimate from conditional analysis; SE Joint, SE for the beta estimate from conditional analysis; P-Joint, conditional analysis P-value having conditioned with all independent variants at the locus. Previously unreported loci are highlighted in bold. All conditionally independent variants were present in at least 50% of the maximum sample size in the European meta-analysis and passed the genome-wide significant threshold (P-Joint < 5 × 10-8) in conditional analysis.

File Name: Supplementary Data 7
Description: **Loci associated with JT. A) Previously not reported loci B) Significant known loci (Reported for multi-ancestry meta-analysis only).** Summary statistics shown for lead variants at independent loci for each genome-wide association study meta-analysis. Study level linear regression summary statistics (two-sided) were meta-analysed using METAL, where effect size estimates were weighted using the inverse of the corresponding standard errors. A Bonferroni-corrected threshold for multiple testing ($P<5\times10^{-8}$) was used to declare genome-wide significance. CHRPOSID, Unique variant identifier; Chr, chromosome; Position, base pair position in build 37; EA, Effect allele; OA, Other allele; EAF, Effect allele frequency; Beta, Effect estimate from linear regression (inverse normal transformed phenotype); SE, Standard error; P-value, Beta (ms), corresponding effect size estimate from untransformed meta-analysis; Het I2, Heterogeneity I2 statistic; P-value of association; *result from X-chromosome male stratified analysis, †locus significant in ancestry-specific analysis but not in multi-ancestry meta-analysis.

File Name: Supplementary Data 8
Description: **Loci associated with QRS. A) Previously not reported loci B) Significant known loci (Reported for multi-ancestry meta-analysis only).** Summary statistics shown for lead variants at independent loci for each genome-wide association study meta-analysis. Study level linear regression summary statistics (two-sided) were meta-analysed using METAL, where effect size estimates were weighted using the inverse of the corresponding standard errors. A Bonferroni-corrected threshold for multiple testing ($P<5\times10^{-8}$) was used to declare genome-wide significance. CHRPOSID, Unique variant identifier; Chr, chromosome; Position, base pair position in build 37; EA, Effect allele; OA, Other allele; EAF, Effect allele frequency; Beta, Effect estimate from linear regression (inverse normal transformed phenotype); SE, Standard error; P-value, Beta (ms), corresponding effect size estimate from untransformed meta-analysis; Het I2, Heterogeneity I2 statistic; P-value of association; *result from X-chromosome male stratified analysis, †locus significant in ancestry-specific analysis but not in multi-ancestry meta-analysis.

File Name: Supplementary Data 9
Description: **Overlap of loci between JT and QRS multi-ancestry meta-analyses where a lead variant in one analysis was also genome-wide significant in the other.** Comparison of multi-ancestry summary statistics for lead variants at independent loci where overlap between JT and QRS GWAS meta-analyses was observed. Study level linear regression summary statistics (two-sided) were meta-analysed using METAL, where effect size estimates were weighted using the inverse of the corresponding standard errors. A Bonferroni-corrected threshold for multiple testing ($P<5\times10^{-8}$) was used to declare genome-wide significance. CHRPOSID, Unique variant identifier; Chr, chromosome; Position, base pair position

in build 37; EA, Effect allele; Beta, Effect estimate from linear regression (inverse normal transformed phenotype); P-value, P-value of association in the respective GWAS meta-analysis. Lead variant for JT on the left panels with look up of corresponding variant in QRS analysis. Right panels show lead variants for QRS with corresponding look up of the variant in the JT analysis. Sections in bold indicate loci which are previously unreported for the trait.

File Name: Supplementary Data 10
Description: **Loci which overlap across QT, JT, QRS, PR and heart rate.** Chr, chromosome; Position, base pair position in build 37; Candidate gene, most likely gene; Distance (kb), Distance of lead variant for each ECG trait from the QT interval lead variant at each locus (kb); r2, correlation with lead QT variant. Overlap was declared if lead variants were within r2 0.1 or within +/- 500kb.

File Name: Supplementary Data 11
Description: **Gene-based analyses (for QT, JT and QRS) conditioning on common variant(s) in the region in 76,202 individuals from the UK Biobank.** N, total sample size from UK Biobank; No. of variants, Number of rare variants included in burden testing; P-value unconditional, Gene-based P-value for association with the ECG trait using Sequence Kernel Association Testing in rareMETALs; P-Conditioning on coding variant within gene; Sequence Kernel Association Testing P-value after conditioning on the rare coding variant with the smallest P-value (details of variant in the row below); P-Conditioning on low frequency/common variant(s) in locus*, Sequence Kernel Association Testing P-value after conditioning on low/common variants at the locus as identified from the corresponding European-ancestry single variant GWAS meta-analysis. N/A, There was no genome-wide significant common/low frequency variants in the single variant meta-analysis at this locus.

File Name: Supplementary Data 12a
Description: **Variant effect predictor results for QT JT and QRS loci from multi-ancestry meta-analyses.** Lead variant rsID, Lead variant at the locus; Chr, chromosome; Position, base pair position in build 37; Analyzed variant rsID, Variant (either lead or proxy (r2 > 0.8) annotated by VEP; r2, Correlation between analysed and lead variant at the locus; HGVS, Human Genome Variation Society; AF, Allele Frequency; All lead variants and their proxies (r2 > 0.8) were annotated using Variant Effect Predictor (VEP). Only variants predicted to be protein altering (determined as high or moderate impact by VEP) are shown. Variants at previously unreported loci are highlighted in bold.

File Name: Supplementary Data 12b
Description: **Annotation of variants with Combined Annotation Dependent Depletion (CADD scores).** CADD scores for variants ranked filtered to a CADD score > 10. Rows in bold represent variants at previously unreported loci for that trait. Lead variant rsID, Lead variant at the locus; Analyzed variant rsID, Variant annotated using CADD; Chr, chromosome; Position, base pair position in build 37; EA, Effect allele; Consequence, Predicted effect of the EA as annotated by Variant Effect PRedictor; r2, linkage disequilibrium r2 between lead variant at locus and annotated variant; MAF, minor allele frequency; CADD, Combined Annotation Dependent Depletion score

File Name: Supplementary Data 13

Description: **Significant cis-eQTLs for lead variants or their proxies (r2>0.8) from GTEx version 8 in heart (left ventricle (LV) and right atrial appendage (RAA)) and brain tissues.** Chr: chromosome; Position: physical position of the lead variant (build 37), EA/OA, Effect / Other Allele; r2: linkage equilibrium r2 between the lead variant (rsID) and proxy eQTL variant(s); GWAS beta, effect size from corresponding inverse normal transformed phenotype multi-ancestry meta-analysis; GWAS P-value, P-value from genome-wide association study meta-analysis; Gene(eQTL): gene from cis-eQTL results; eQTL Beta, Normalized effect size on gene expression at that the corresponding tissue; PP, posterior probability of a common causal variant (>0.75 was set as significance threshold) calculated using the COLOC package in R (methods) - Rows in bold are where this condition is met. eQTL: Expression quantitative trait locus.

File Name: Supplementary Data 14a
Description: **Hi-C results for lead variants and proxies (r2 > 0.8) from the multi-ancestry meta-analysis using Fit-Hi-C pipeline.** Results shown for variants with RDB score ≤ 3b. r2: correlation (r2) between the lead variant (rsID) and analysed variant(s); RDB score, Regulome DB score; NA, No significant result in that tissue

File Name: Supplementary Data 14b
Description: **Promoter-capture Hi-C results for lead variants and proxies (r2 > 0.8) from the multi-ancestry meta-analysis.** Promoter interactions as defined by Jung et al, were identified for all lead variants and their proxies (r2 > 0.8). Results were filtered to only include those variants with a RDB score <= 3b. Results shown for variants with RDB score <= 3b. r2: correlation (r2) between the lead variant (rsID) and analysed variant(s); RDB score, Regulome DB score

File Name: Supplementary Data 15
Description: **Gene-set tissue/cell type enrichment using Data driven Expression-Prioritization Integration for Complex Traits (DEPICT) for QT, JT and QRS multi-ancestry meta-analyses.** MeSH term, Medical Subject Heading term for the tissue or cell type annotation; Name, Tissue or cell type annotation; MeSH first level term, Description of the tissue or cell type annotation; MeSH second level term, More general description of the tissue or cell type annotation; Nominal P-value, Nominal enrichment P-value of tissue/cell type annotation from the analysis as output from DEPICT by comparing z-scores from Welch's t-test against the null hypothesis (Genes in associated loci are not highly expressed in the given tissue or cell type); FDR, False discovery rate; Z score, Tissue-specific expression Z-score for enrichment. Results are shown if they met a false discovery rate (FDR) < 0.01 for statistical significance.

File Name: Supplementary Data 16
Description: **DEPICT pathway gene-set enrichment results for QT, JT and QRS multi-ancestry meta-analysis.** GO, Gene ontology; KEGG, Kyoto encyclopedia of genes and genomes; Original gene set ID, Identifier of the predefined gene set; Original gene set description, Description of the predefined gene set; Nominal enrichment P-value of the reconstituted gene set from the meta-analysis result as output by DEPICT, calculated by comparing z-scores from Welch's t-test against the null; FDR, False discovery rate; Z score, Reconstituted gene set Z score. Results are shown for GO categories, mouse knockout phenotypes, protein-protein interaction subnetworks, KEGG and REACTOME pathways separately with

a false discovery rate <0.01 from the analysis being used to declare statistical significance. Results are ordered by increasing P-value.

File Name: Supplementary Data 17
Description: **Literature review of genes indicated by bioinformatic analyses and nearest gene to lead variant for QT.** Literature review of genes indicated by bioinformatic analyses at previous unreported loci only. Likely candidate gene at locus was determined through systematic prioritization weighted in the following order: 1. Non-synonymous variant (with greater weighting when predicted to be damaging or deleterious), 2. Gene with support for eQTL colocalization, 3. Gene prioritized by DEPICT, 4. Gene in HiC interaction, 5. Gene within LD block (r2 > 0.5), 6. Expression in heart (proteinatlas.org), 7. Gene with relevant Mendelian disease, 8. Gene with mouse phenotype, 9. Literature review. Genes with multiple lines of evidence were prioritized by summing the weights and the likely candidate gene chosen as the one with the highest score. Where there were multiple genes with the same score, all are included.

File Name: Supplementary Data 18
Description: **Literature review of genes indicated by bioinformatic analyses and nearest gene to lead variant for JT.** Literature review of genes indicated by bioinformatic analyses at previous unreported loci only. Likely candidate gene at locus was determined through systematic prioritization weighted in the following order: 1. Non-synonymous variant (with greater weighting when predicted to be damaging or deleterious), 2. Gene with support for eQTL colocalization, 3. Gene prioritized by DEPICT, 4. Gene in HiC interaction, 5. Gene within LD block (r2 > 0.5), 6. Expression in heart (proteinatlas.org), 7. Gene with relevant Mendelian disease, 8. Gene with mouse phenotype, 9. Literature review. Genes with multiple lines of evidence were prioritized by summing the weights and the likely candidate gene chosen as the one with the highest score. Where there were multiple genes with the same score, all are included.

File Name: Supplementary Data 19
Description: **Literature review of genes indicated by bioinformatic analyses and nearest gene to lead variant for QRS.** Literature review of genes indicated by bioinformatic analyses at previous unreported loci only. Likely candidate gene at locus was determined through systematic prioritization weighted in the following order: 1. Non-synonymous variant (with greater weighting when predicted to be damaging or deleterious), 2. Gene with support for eQTL colocalization, 3. Gene prioritized by DEPICT, 4. Gene in HiC interaction, 5. Gene within LD block (r2 > 0.5), 6. Expression in heart (proteinatlas.org), 7. Gene with relevant Mendelian disease, 8. Gene with mouse phenotype, 9. Literature review. Genes with multiple lines of evidence were prioritized by summing the weights and the likely candidate gene chosen as the one with the highest score. Where there were multiple genes with the same score, all are included.

File Name: Supplementary Data 20
Description: **Potential drug targets from the druggable gene set database.** Druggability tier: Categorization as per druggable genome database (Finan et al, PMID 28356508) (methods). ADME gene: Is the protein product of the gene involved in absorption, distribution, metabolism, and excretion (ADME) of a compound. Known targets of class I, II, III and IV anti-arrhythmic drugs were identified using the KEGG drug database and excluded from the list (4 for QT - *KCNH2*, *KCNQ1*, *SCN5A*, *SCN10A*, 6 for JT - *CACNA1D*, *KCND3*, *KCNH2*, *KCNQ1*, *SCN5A*, *SCN10A* and 5 for QRS - *CACNA1D*, *KCND3*, *KCNQ1*, *SCN5A*, *SCN10A*)Supplementary Data 21: Association of genetically determined QT, JT and QRS in unrelated European individuals from UK Biobank

File Name: Supplementary Data 21
Description: **Association of genetically determined QT, JT and QRS in unrelated European individuals from UK Biobank.** Findings from the logistic regression analysis for each polygenic risk score and clinical outcome. Units are per standard deviation increase in polygenic risk score. SE; standard error, OR; Odds ratio for association, CI: Confidence interval, AVB; Atrioventricular block, PPM; Pacemaker implantation, BBB; Bundle branch block. OR columns are colour coded according to direction of effect. P-values <0.05 are in bold and red. A Bonferroni threshold for statistical significance was used to account for multiple testing (0.05/8 = 6.3x10-3).

File Name: Supplementary Data 22
Description: **Study information for sudden cardiac death cohorts.** No.variants included in PRS; Number of lead variants at loci in the European-ancestry meta-analysis for each ECG trait, which passed the imputation quality threshold (Rsq > 0.8) and were thus included in the model

File Name: Supplementary Data 23
Description: **Meta-analysis results for PRS association with SCD results for each trait.** Results for the meta-analysis of per-study summary statistics for association of each PRS with sudden cardiac death, using R-package Meta (v5.5.0) and an inverse-variance weighted fixed effects model. Effect; Increase in risk of SCD per 1 millisecond increase in the average ms per allele, StdErr; Standard Error; Direction; Direction of effect of each study in the following order ARIC-CABS-FinGesture (and NFBC1966). Significant result (P-value < 0.05) is in bold.

File Name: Supplementary Data 24
Description: **Summary of all input data and tools used for each analysis.** GWAS, Genome-wide association study; N/A, Not applicable for this analysis; 1000G, 1000 genomes; EST-UKB, Exercise stress test UK Biobank cohort; SNP, Single nucleotide polymorphism; eQTL, expression Quantitative Trait Locus

File Name: Supplementary Data 25
Description: **Codes used to define each clinical outcome in the UK.** ICD10, International Classification of Diseases (ICD) 10th Revision code; ICD9, International Classification of Diseases (ICD) 9th Revision code, OPCS4, OPSC classification of interventions and procedures (version 4).
